# Identification and characterization of novel endolysins targeting *Gardnerella vaginalis* biofilms to treat bacterial vaginosis

**DOI:** 10.1038/s41522-022-00285-0

**Published:** 2022-04-19

**Authors:** Sara Arroyo-Moreno, Matthew Cummings, David B. Corcoran, Aidan Coffey, Ronan R. McCarthy

**Affiliations:** 1 Division of Biosciences, Department of Life Sciences, College of Health and Life Sciences, Brunel University London, Uxbridge, UB8 3PH UK; 2 Department of Biological Sciences, Munster Technological University, Cork, Ireland; 3 CC Biotech Ltd, London, UK

**Keywords:** Biofilms, Antimicrobials, Pathogens, Microbial communities

## Abstract

Bacterial vaginosis (BV) is a recurrent dysbiosis that is frequently associated with preterm birth, increased risk for acquisition of human immunodeficiency virus (HIV) and other sexually transmitted infections (STIs). The overgrowth of a key pathobiont, *Gardnerella vaginalis*, as a recalcitrant biofilm is central to the development of this dysbiosis. Overgrowth of vaginal biofilms, seeded by initial *G. vaginalis* colonization, leads to recurrent symptomatic BV which is poorly resolved by classically used antibiotics. In this light, the use of bacteriophages and/or their proteins, represents a promising alternative. Here we identify 84 diverse anti-*Gardnerella* endolysins across 7 protein families. A subset of 36 endolysin candidates were refactored and overexpressed in an *E. coli* BL21 (DE3) system and 5 biochemically and structurally diverse endolysins were fully characterized. Each candidate endolysin showed good lytic activity against planktonic *G. vaginalis* ATCC14018, as well as *G. vaginalis* clinical isolates. These endolysin candidates were assayed in biofilm prevention and disruption assays, with biofilm disruption at low microgram concentrations (5 μg/ml) observed. In addition to clonal *G. vaginalis* biofilms, endolysin candidates could also successfully disrupt polyspecies biofilms. Importantly, none of our candidates showed lytic activity against commensal lactobacilli present in the vaginal microbiota such as *L. crispatus, L. jensenii*, *L. gasseri*, and *L. iners* or against *Atopobium vaginae* (currently classified as *Fannyhessa vaginae*). The potency and selectivity of these novel endolysins constitute a promising alternative treatment to combat BV, avoiding problems associated with antibiotic resistance, while retaining beneficial commensal bacteria in the vaginal flora. The diverse library of candidates reported here represents a strong repository of endolysins for further preclinical development.

## Introduction

Bacterial vaginosis (BV) is the most common vaginal dysbiosis in women of reproductive age with a prevalence of 10–30% worldwide and is the cause of several serious sequelae for patients^1,2^. Approximately one in six patients experiences symptomatic BV, which involves a malodorous vaginal discharge. BV is often highly psychologically distressing to the patient and the prenatal health risks for both symptomatic and asymptomatic patients are concerning^3^. All BV carriers face increased likelihood of acquisition and transmission of the human immunodeficiency virus, preterm birth, spontaneous abortion, pelvic inflammatory disease, and postoperative infection^2^. A primary pathobiont associated with BV is the opportunistic pathogen, *Gardnerella vaginalis* being isolated in up to 95% cases^4^.

*G. vaginalis* is a Gram-variable facultative anaerobic bacterium that is commonly present in low numbers in the vaginal flora of healthy women^5^. The healthy vaginal epithelium is generally dominated by hydrogen peroxide and lactic acid producing lactobacilli, which acidify the vaginal environment and limit the adhesion and growth of key pathobionts^6^. If the healthy composition of the vaginal flora is perturbed, anaerobic opportunistic pathogens such as *G. vaginalis* can proliferate leading to development of bacterial vaginosis. *G. vaginalis* possesses a range of virulence factors that supports its ability to colonize and persist in the vaginal microenvironment, particularly its biofilm-forming capacity and its cytotoxicity, which is associated with the production of vaginolysin, prolidase and sialidase^7^*. G. vaginalis* can be considered a seeding species, where initial biofilm formation by this pathogen on the vaginal epithelium facilitates the attachment and proliferation of other BV associated pathogens, such as *Atopobium vaginae* (currently classified as *Fannyhessa vaginae*). This results in the formation of a polymicrobial biofilm that, based on clinical and in vitro evidence, is only weakly affected by the innate immune response or antibiotic therapy^8–10^.

Bacterial biofilms are associated with both the initial development and recurrence of BV. In vivo studies revealed that after successful therapy with oral metronidazole (the clinical treatment of choice for BV), patches of biofilms consisting of *G. vaginalis* and *A. vaginae* persisted on vaginal epithelial cells^2,11^. Poor penetration of *G. vaginalis* biofilms by metronidazole (and other antibiotics, such as clindamycin) has been associated with a high rate of recurrence, with up to 60% of women experiencing a second presentation of BV within 12 months of initial treatment. This leads to a consistent cycle of treatment using inefficient antimicrobials in the clinic, generating an increasingly resistant target^12^.

Clearly, more efficacious treatments of BV are required in the clinic, particularly those capable of disrupting the biofilms fundamental to the development and recurrence of this dysbiosis. One promising alternative to treat BV may be found from bacteriophage (phage) encoded peptidoglycan hydrolases^13^. Phage generally encode two different types of peptidoglycan hydrolases: tail-associated hydrolases and endolysins. Recombinant versions of both represent promising alternatives to classic antibiotics for a variety of human pathogens^14^. *G. vaginalis* represents a tractable target for these phage-derived enzymes as it does not have an outer membrane, leaving its peptidoglycan cell wall exposed and accessible to these phage-encoded peptidoglycan hydrolases^15^. *G. vaginalis* prophage elements are a potential rich source of novel endolysins which are diverse in sequence, structure and endolysin domain composition. A previous report undertaken contemporaneously with this study illustrates the existence of an anti-*Gardnerella* endolysin^16^. Phage-encoded peptidoglycan hydrolases have shown promising efficiency in biofilm disruption^17^ and possess an extremely narrow lytic spectrum (species/strain level specificity is commonly reported). Critically, there has been no evidence reported of bacteria evolving resistance to their activity^18^. In this study, several novel classes of structurally distinct endolysin candidates encoded by *G. vaginalis* prophages were identified in silico. Their protein solubility and stability conditions were then assessed. In total, five endolysin candidates were selected for characterization of bioactivity, selectivity and propensity to drive resistance in clinically relevant *G. vaginalis* isolates.

## Results

### Anti-*Gardnerella* endolysin discovery

**Fig. 1:**
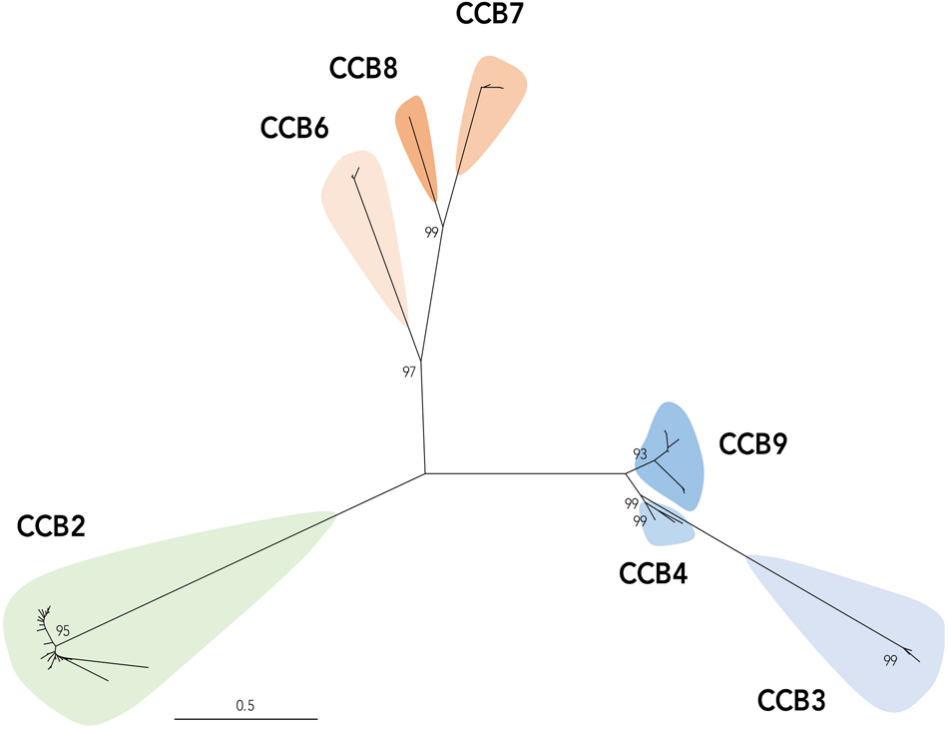
Phylogenetic analysis of anti-Gardnerella endolysin candidate library. Individual families are denoted by coloured clades. Selected bootstrap values of >95% are shown for clarity. CCB2 homologues are described previously^16^.

A previous report undertaken contemporaneously with this study illustrates the existence of an anti-*Gardnerella* endolysin, referred to in this publication as CCB2^16^. This report illustrated bacteriolytic activity of this endolysin against *Gardnerella* spp., and demonstrated the potential for homologous domain swapping to generate structurally similar derivative endolysins of increased potency characteristics. However, a more comprehensive exploration of the full diversity of this medically important and biochemically convergent class of enzyme is desirable. Our in silico analysis of publicly available *Gardnerella* genome sequences identified a library of 84 putative anti-*Gardnerella* endolysins which were categorized into seven diverse endolysin families (CCB2, 3, 4, 6, 7, 8 and 9) (Table 1, Fig. 1). The enzymatic active domain (EAD) of putative endolysins within the library were predominantly members of the glycoside hydrolase 25 family, which cleave the b-1,4-glycosidic bond between N-acetylmuramic acid and N-acetylglucosamine sugars in bacterial peptidoglycan. Two distinct types of GH25 EAD were present in the identified library including LysA-like (CCB3, 6, 7, 8 and 9) and LytC (CCB2). LytC-like GH25 EADs were most frequently identified in this library of putative endolysins, representing 47% of all candidates despite being constrained to the CCB2 endolysin family. LysA-like GH25-containing endolysin candidates represented a marginally smaller proportion of the library but were commonly associated with differing cell wall binding domains. This indicates that this EAD may be more tolerant of natural recombination events, and by extension, rational endolysin engineering strategies. Interestingly, members of the CCB4 family comprise an N-acetylmuramoyl-l-alanine amidase, or amidase_2 EAD, and represent the only endolysin within our library predicted to cleave a different bond within the bacterial peptidoglycan.

**Table 1:** Domain composition of anti-*Gardnerella* endolysin candidates.

Endolysin Family	Endolysin domain type			
	EAD	CWB 1	CWB 2	Schematic
CCB2^a^	Glyco_hydro_25	Cw_7	Cw_7	
CCB3	Amidase_2	RBP-like^b^		
CCB4	Glyco_hydro_25	RBP-like^b^		
CCB6	Glyco_hydro_25	Cw_binding_1		
CCB7	Glyco_hydro_25	SH3b		
CCB8	Glyco_hydro_25	SH3b		
CCB9	Glyco_hydro_25	RBP-like^b^		

^a^Truncated variants comprising a single Cw_7 were commonly observed within CCB2-family endolysins.

^b^Putative in silico characterization of CWB domains as phage receptor binding protein (RBP) or phage baseplate protein homologues. Domain designation for endolysins schematics are as follows: green: glyco_hydro_25, blue: amidase_2, red: Cw_7, yellow: Cw_binding_1, orange: SH3b, and white: RBP-like. Black lines indicate endolysin domain linkers.

The diversity of cell-wall binding (CWB) domains both within and between putative endolysin families is considerably higher than that described for cognate endolysin EADs. CCB2 family endolysins comprised either one or two C-terminal CW_7 CWB repeat domains. It is unclear whether the variation in CWB repeat occurred due to truncation or duplication events in CCB2 homologues. Structurally diverse Cw_binding_1 and SH3b CWB domains were identified in CCB6, and CCB7 and 8 respectively. Whilst CCB7 and 8 both comprise single SH3b domains, amino acid sequence similarity at the domain level between the two families is low (65%). Strikingly, 36% of all putative endolysins lacked canonical or well-defined cell wall binding domains. Further interrogation of the C-terminal region of these candidates often identified structural and/or sequence similarities to phage baseplate or phage receptor binding proteins (RBP). CCB3, CCB4 and CCB9 exemplify possible natural endolysin EAD:phage-derived-host-binding-domain chimera formation. This naturally occurring evolutionary process can now be plausibly mimicked as an engineering strategy to alter endolysin specificity and bioactivity and this represents a logical progression of the exploration of wild-type endolysin domain structures. Genes encoding endolysins were widely distributed throughout the *Gardnerella* Genus. Our analysis identified both prophage elements and endolysins encoded within the genomes of members of 7 of the 9 *Gardnerella* genomospecies (GS01–GS09) proposed by Potter et al. in 2019^19^ (Supplementary Table 1). CCB2 family endolysins were most ubiquitous, but were missing from GS02, GS04 and GS06 group organisms. CCB3 and CCB4 family endolysins were identified in numerous organisms classified as GS01 and GS02, as well as *Gardnerella* species not characterized using this taxonomic method, including *G. vaginalis* FDAARGOS_568. Similarly, CCB6, 7 and 8 group endolysins were isolated from genomes defined as *Gardnerella vaginalis* which fall outside of the genome species taxonomy. Strikingly, identification of CCB9 was restricted to genomes of *Gardnerella* genomospecies GS03. In fact, CCB9 homologues were represented in 71% of GS03 genomes analysed, including *G. swidsinskii* GS 9838-1 but were absent from *G. leopoldii* UGent 06.41, which is also defined as a member of GS03. *Gardnerella* species within the GS04 and GS06 groups lacked any detectable putative endolysin encoding genes. This is in contrast to all other genomes analysed, with the exception of *G. piotii* UGent 18.01, where at least one endolysin per genome was identified. Our analysis of GS04 and GS06 genome species was limited to three and one genome(s), respectively, however, analysis of additional *Gardnerella* genomes from these genomospecies may yield new esoteric endolysin candidates.

### Endolysin stability assessment

A subset of 36 endolysins representing structurally and sequence diverse library members were refactored for expression in *Escherichia coli* BL21(DE3). Each recombinant protein comprised an N-terminal hexa-histidine tag to aid identification and purification. Extraction of recombinant proteins in PBS buffer systems at a neutral pH was met with mixed success. For example, CCB2M84_97 of the CCB2 family formed inclusion bodies when produced as described in 4.1, despite showing 91.2% AA sequence similarity to CCB2M87_2 which showed good solubility (Supplementary Fig. 2). Further analysis of multiple additional CCB2 family members highlighted two amino acid residues, A53T and N233S, which were common to all soluble members. Homology models constructed for each endolysin domain showed both residues to be solvent exposed, with the latter positioned at the C-terminus of the flexible linker between the EAD and first CWB (Supplementary Fig. 3).

To systematically improve long-term protein stability and circumvent any problematic endolysin solubility concerns, a subset of 10 endolysin candidates (Table 2) were subject to thermal shift analysis across 288 individual buffer conditions comprising alternate buffering agents, osmolytes and salts. Most endolysin candidates showed improved thermal stability in 2-(*N*-morpholino) ethanesulfonic acid (MES) buffer (pH 5.6) (average *T*_m_ improvement of 17.5 °C over Table 2 buffer), followed by citrate (pH 4.1) and acetic acid (pH 4.7) based buffers (Supplementary Fig. 4). Based on stability and yield, a selection of five structurally and biochemically diverse endolysin candidates were chosen for bioactivity characterization (Supplementary Table 2).

**Table 2:** Size exclusion chromatography buffers used for the different endolysins.

Recombinant endolysin	SEC buffer
CCB2M94_8	50 mM HEPES, 300 mM NaCl, 1 mM TCEP, 20% glycerol pH 7
CCB2.2	20 mM Tris, 300 mM NaCl, 1 mM TCEP, 10% glycerol, 0.05% Tween 20, pH 8.5
CCB2.4	20 mM Tris, 300 mM NaCl, 1 mM TCEP, 10% glycerol, 0.05% Tween 20, pH 8.5
CCB230b	PBS, 300 mM NaCl, 1 mM TCEP, 20% glycerol pH 7.4
CCB3.2	20 mM Tris, 300 mM NaCl, 1 mM TCEP, 10% glycerol, 50 mM l-arginine, 50 mM glutamic acid, pH 8.5
CCB4.1	PBS, 300 mM NaCl, 1 mM TCEP, 20% glycerol pH 7.4
CCB4.2	PBS, 300 mM NaCl, 1 mM TCEP, 20% glycerol pH 7.4
CCB7.1	20 mM Tris, 300 mM NaCl, 1 mM TCEP, 10% glycerol, 0.05% Tween 20, pH 8
CCB8.1	50 mM HEPES, 500 mM NaCl, 1 mM TCEP, 10% glycerol pH 7.4
CCB2M90_2	20 mM sodium phosphate, 200 mM NaCl, 1 mM TCEP, 10 % glycerol, pH 8.0

### MIC assays and specificity of endolysin candidates

The MIC of the different endolysin candidates was assessed against *G. vaginalis* ATCC14018, *G. vaginalis* UG860107 and *G. vaginalis* BUL001. Endolysins CCB2M94_8, CCB7.1, CCB8.1, CCB2.2 and CCB4.1 presented an MIC of ≤100 µg/ml (Table 3). Due to their potent activity, these candidates were selected for further analyses. The endolysin candidate CCB2M94_8 was published while this study was being undertaken^16^, however we report it here as it represents an excellent internal control. MIC values for the different endolysin candidates were observed to be similar for the three different *G. vaginalis* strains. CCB8.1 showed the lowest MIC values for all of the strains tested (25 μg/ml), presenting even lower values than the frontline BV antibiotics such as metronidazole and clindamycin against clinical isolates (Table 3). CCB2M94_8, CCB7.1 and CCB2.2 presented MIC values similar to those observed for metronidazole and clindamycin against clinical isolates.

**Table 3:** MIC for five different endolysin candidates, metronidazole, and clindamycin against three different *G. vaginalis* strains.

Endolysin/antibiotic	MIC (μg/ml)		
	*G. vaginalis* ATCC14018	*G. vaginalis* UG860107	*G. vaginalis* BUL001
CCB2M94_8	100	100	100
CCB7.1	100	100	100
CCB8.1	25	25	25
CCB2.2	100	50	100
CCB4.1	100	100	100
Metronidazole	12.5	100	100
Clindamycin	0.78	50	50

To determine their range of activity, the five selected endolysin candidates were also assessed for MIC against common commensal vaginal lactobacilli including *L. jensenii*, *L. gasseri*, *L. crispatus and L. iners* (Table 4). None of the endolysin candidates could produce growth inhibition against *Lactobacillus* at the range of tested enzymatic concentrations. The same effect was observed for metronidazole, although the tested vaginal bacteria presented susceptibility against clindamycin. This demonstrated the anticipated narrow spectrum of activity of these candidates, highlighting their potential to selectively target and eradicate a key BV pathobiont while leaving the commensal community intact.

**Table 4:** MIC for five different endolysin candidates, metronidazole and clindamycin against vaginal lactobacilli and *A. vaginae*.

Endolysin/antibiotic	MIC (μg/ml)				
	*L. jensenii* CCUG35572	*L. gasseri* CCUG4409	*L. crispatus* CCUG42898	*L. iners* CCUG38673	*A. vaginae* UG71161
CCB2M94_8	≥200	≥200	≥200	≥200	≥200
CCB7.1	≥200	≥200	≥200	≥200	≥200
CCB8.1	≥200	≥200	≥200	≥200	≥200
CCB2.2	≥200	≥200	≥200	≥200	≥200
CCB4.1	≥200	≥200	≥200	≥200	≥200
Metronidazole	≥200	≥200	≥200	≥200	≥200
Clindamycin	200	200	0.39	1.56	50

### Resistance assays

While to date, there has been no evidence of the evolution of resistance to endolysins^9^, it is still an important consideration when developing novel therapeutics. To test this among our five endolysin candidates, *G. vaginalis* ATCC14018 was cultured using increasing concentrations of the five selected endolysin candidates in an attempt to drive the evolution of resistant clones. After five serial passages, no resistant colonies were isolated suggesting that these endolysins conform to the established paradigm that the evolution of resistance to the activity of endolysins is currently undetermined.

### *G. vaginalis* biofilm prevention

**Fig. 2:**
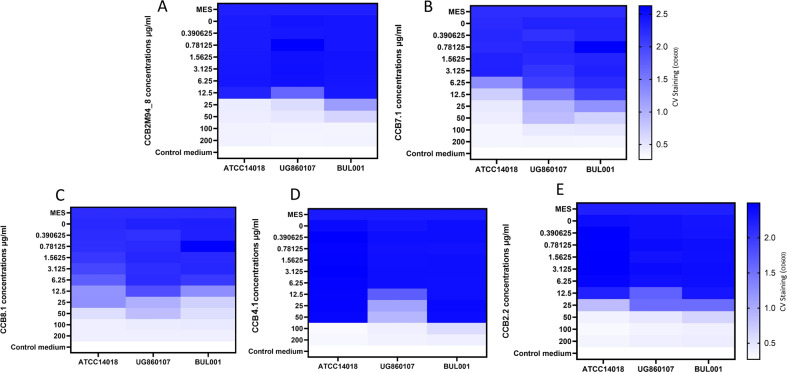
Endolysin biofilm prevention activity: Biofilm prevention assay using G. vaginalis ATCC14018, G. vaginalis UG860107, and G. vaginalis BUL001 cultures. Final concentrations of **A** CCB2M94_8, **B** CCB7.1, **C** CCB8.1, **D** CCB4.1 or **E** CCB2.2, from 0.39 to 200 μg/ml were used to prevent biofilms. OD_600_ readings are the average of biological triplicates. Controls: MES corresponds to bacteria with MES buffer and control medium is the no bacterial control.

**Fig. 3:**
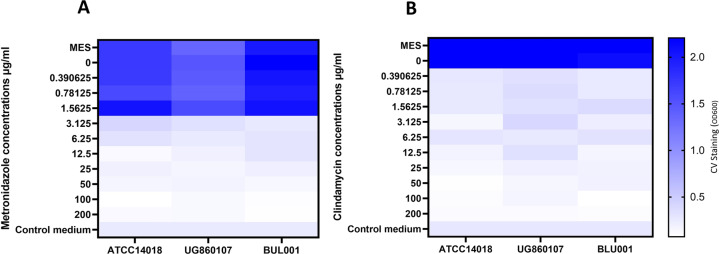
Antibiotic biofilm prevention activity: Biofilm prevention assays using *G. vaginalis* ATCC14018, *G. vaginalis* UG860107 and *G. vaginalis* BUL001 cultures. Final concentrations of **A** metronidazole or **B** clindamycin from 0.39 to 200 μg/ml, were used to disrupt biofilms. OD_600_ readings are the average of biological triplicates. Controls: MES corresponds to bacteria with MES buffer and control medium is the no bacterial control.

One of the primary clinical complications associated with the eradication of *G. vaginalis* is the capacity of this pathobiont to establish robust biofilms, leading to BV relapse. These biofilms minimize the efficacy of traditional antibiotics while maximizing the probability of treatment failure or infection reoccurrence. The capacity of endolysins to prevent biofilm formation is not fully understood, but is a key consideration when evaluating their efficacy as a BV therapeutic agent. The ability of these endolysins to prevent *Gardnerella* biofilms was determined (Fig. 2, Supplementary Fig. 5). In terms of biofilm prevention, metronidazole and clindamycin presented a higher efficacy compared to the endolysin candidates (Fig. 3, Supplementary Fig. 6), which is unsurprising given their potent activity against planktonic cells. The best performing endolysin candidates were CCB2M94_8 and CCB7.1. For some endolysin candidates, results differed between the tested *G. vaginalis* strains. This could be due to strain-specific differences in the composition of the extracellular matrix of the biofilm.

### Monospecies and polyspecies biofilm disruption

#### *G. vaginalis* monospecies biofilm disruption

**Fig. 4:**
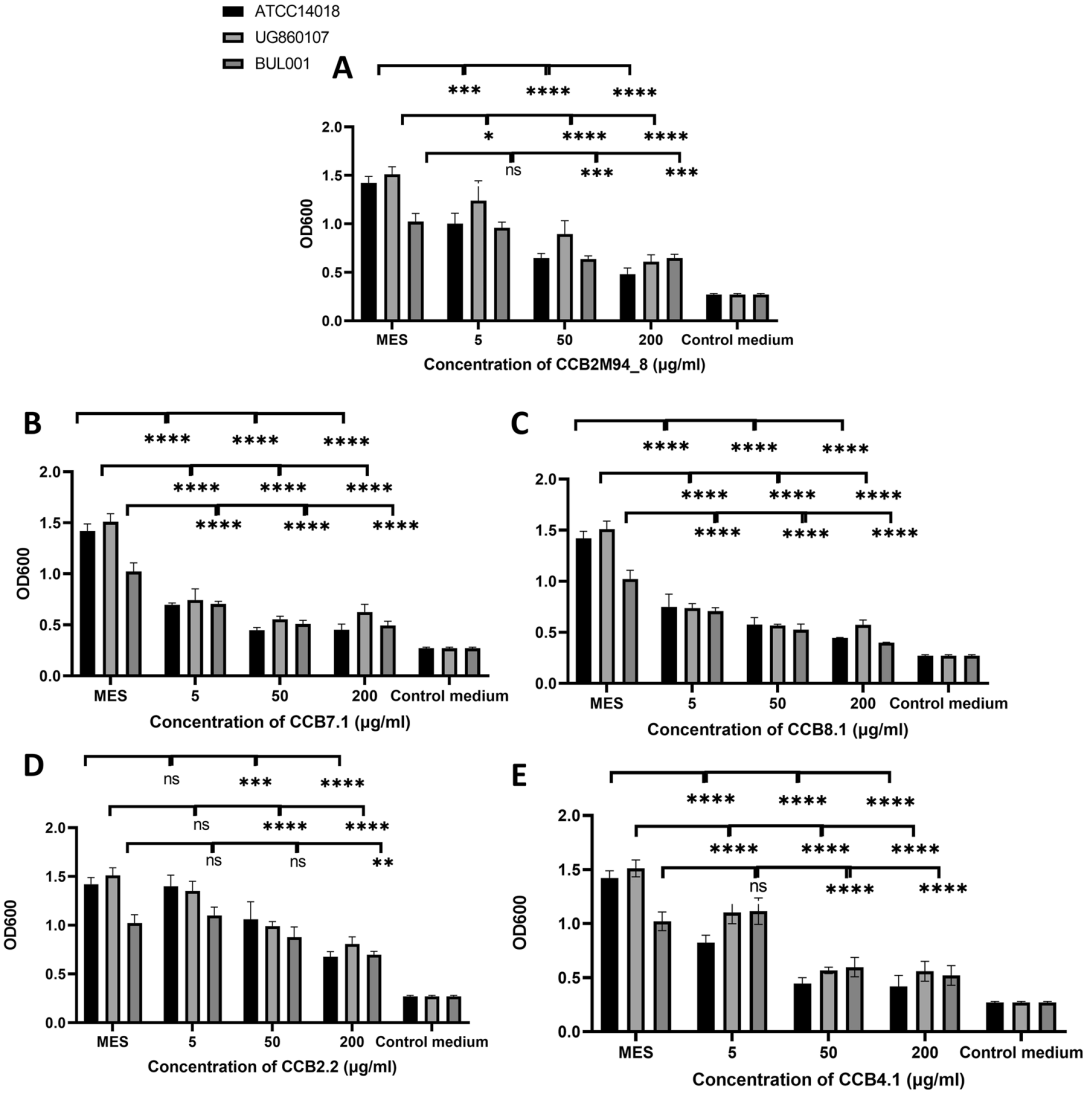
Endolysin biofilm disruption activity*:* Biofilm disruption assay using *G. vaginalis* ATCC14018, *G. vaginalis* UG860107 and *G. vaginalis* BUL001 biofilms. Final concentrations of **A** CCB2M94_8, **B** CCB7.1, **C** CCB8.1, **D** CCB2.2 or **E** CCB4.1, from 5 to 200 μg/ml were used to disrupt biofilms. OD_600_ readings are the average of biological triplicates plus/minus their standard deviation. The asterisks in figures indicate levels of significance. *p*-values < 0.05 are represented by *, *p-*values *<* 0.01 are represented by **, *p*-values < 0.001 are represented by ***, and *p*-values < 0.0001 are represented by ****. No statistical differences are represented by ns. Controls: MES corresponds to bacteria with MES buffer and control medium is the no bacterial control. *p*-values for ANOVA and Tukey tests.

**Fig. 5:**
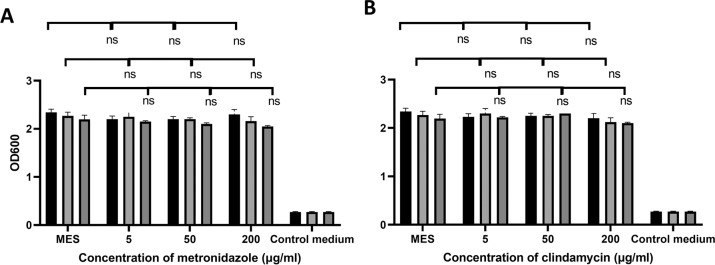
Antibiotic biofilm disruption activity*:* Pre- formed biofilm disruption assays using *G. vaginalis* ATCC14018, *G. vaginalis* UG860107 and *G. vaginalis* BUL001 biofilms. Final concentrations of **A** metronidazole or **B** clindamycin from 5 to 200 μg/ml, were used to disrupt biofilms. OD_600_ readings are the average of biological triplicates plus/minus their standard deviation. No statistical differences are represented by ns. Controls: MES corresponds to bacteria with MES buffer and control medium is the no bacterial control. *p*-values for ANOVA and Tukey tests.

**Fig. 6:**
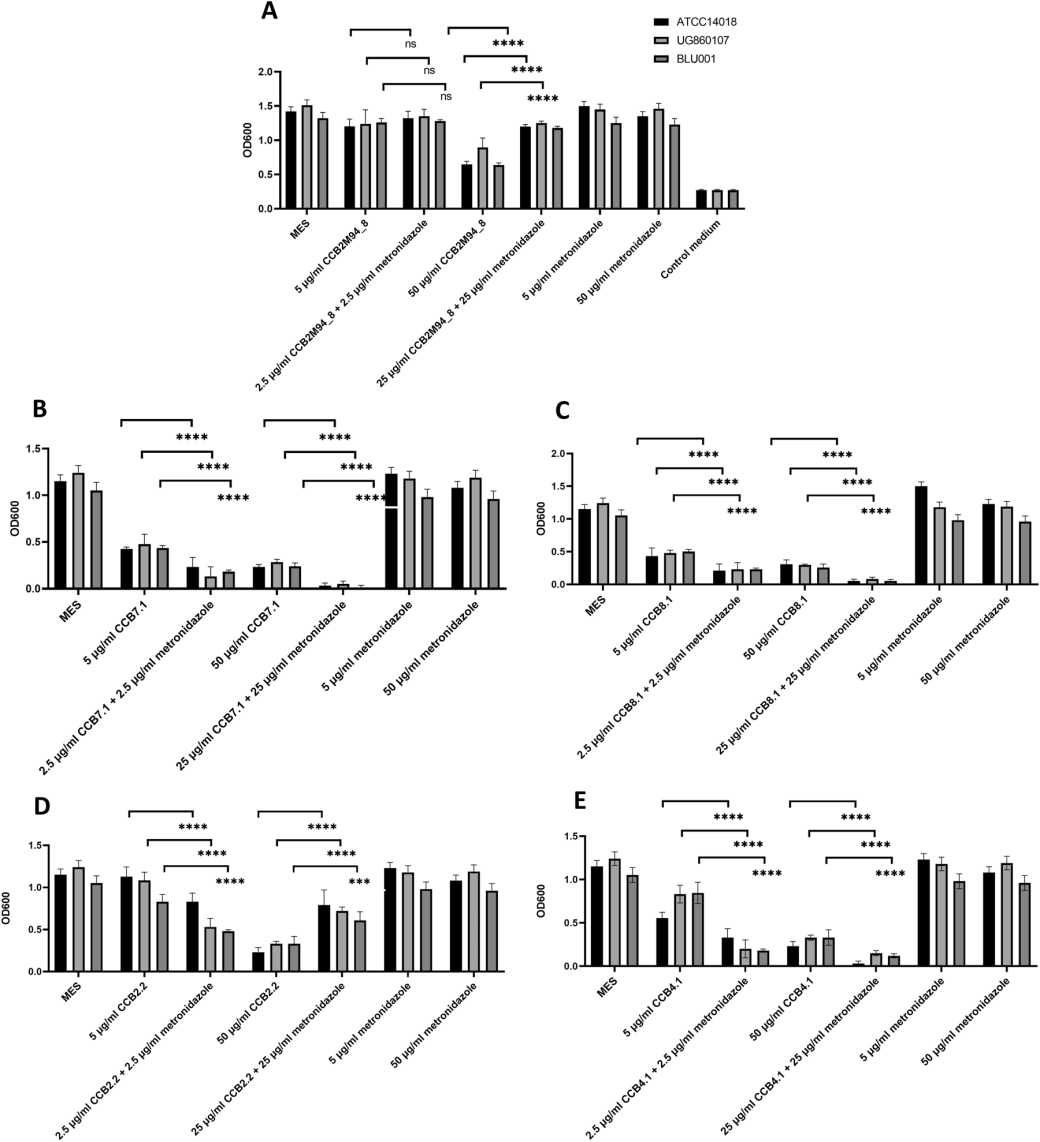
Synergistic activity*:* Biofilm disruption assay using *G. vaginalis* ATCC14018, *G. vaginalis* UG860107 and *G. vaginalis* BUL001 pre-established biofilms. Final concentrations of **A** CCB2M94_8, **B** CCB7.1, **C** CCB8.1, **D** CCB2.2 or **E** CCB4.1 ranged from 2.5 to 50 μg/ml, were used to disrupt biofilms. OD_600_ readings are the average of biological triplicates plus/minus their standard deviation. The asterisks in figures indicate levels of significance. *p*-values < 0.05 are represented by *, *p-*values < 0.01 are represented by **, *p*-values < 0.001 are represented by *** and *p*-values < 0.0001 are represented by ****. No statistical differences are represented by ns. Controls: MES corresponds to bacteria with MES buffer and control medium is the no bacterial control. *p*-values for ANOVA and Tukey tests.

While preventing biofilm formation represents a desirable characteristic for a BV prophylactic, the capacity of our candidate endolysins to disrupt an established biofilm represents an even more important consideration. This is because by the time a patient presents in the clinic with BV, a robust *G. vaginalis* biofilm is likely well established^18^. The five selected endolysin candidates were therefore tested in biofilm disruption assays, to determine their capacity to disrupt a pre-established biofilm. All of the endolysin candidates produced significant biofilm disruption against the three *G. vaginalis* strains at concentrations of 50 and 200 µg/ml (except for CCB2.2 at 50 µg/ml). CCB7.1 and CCB8.1 produced statistically significant biofilm reduction at only 5 µg/ml against all three *G. vaginalis* strains used (Fig. 4). As anticipated, no significant biofilm disruption was observed when metronidazole and clindamycin were employed at concentrations of 5, 50 and 200 µg/ml (Fig. 5) suggesting a likely mechanism for the routine failure of these treatment regimes in the clinic. To determine their ability to synergistically disrupt biofilms when combined with these antibiotics, the endolysin candidates were also used in combination with metronidazole (Fig. 6). Combinations of the endolysin candidates with metronidazole, resulted in significant biofilm disruption for each of the endolysins compared to either the antibiotic or endolysin alone, except for CCB2M94_8. The potential synergistic activity of these endolysins suggests that when used in combination with frontline BV antibiotics, these candidates could help overcome the inefficacy of antibiotics alone with respect to biofilm-associated BV infections.

#### *Gardnerella* dual-species biofilm disruption

**Fig. 7:**
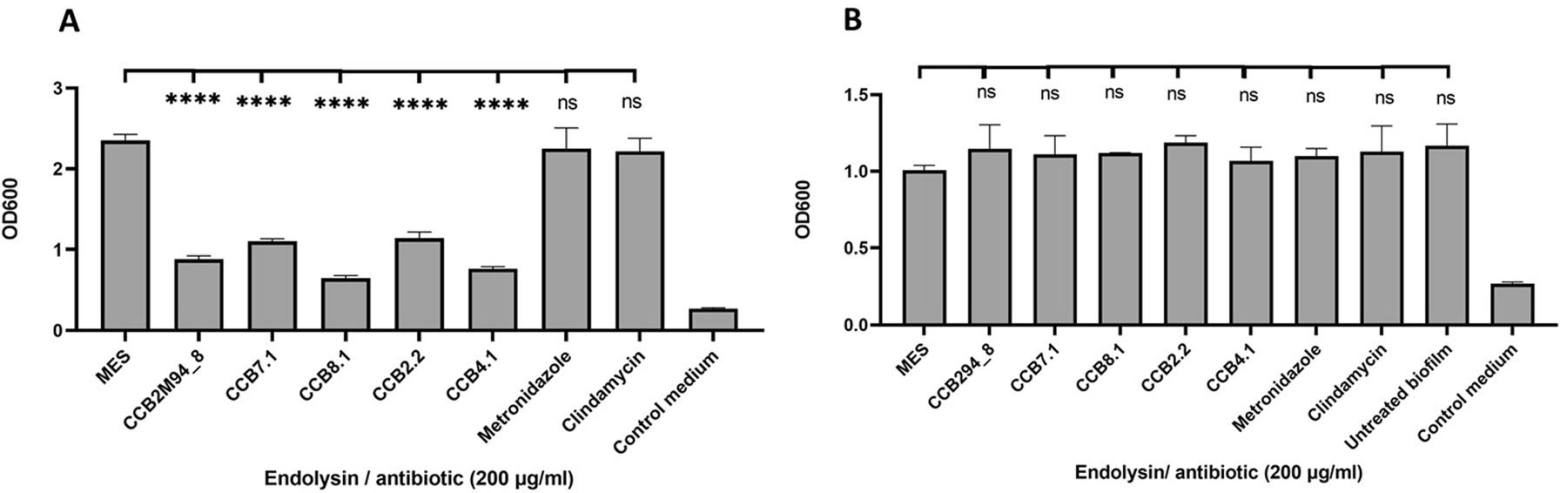
Polymicrobial biofilm disruption. **A** Biofilm disruption assay using *G. vaginalis* ATCC14018 and *A. vaginae* UG71161 polymicrobial biofilms. The different endolysin candidates, metronidazole and clindamycin were used in a final concentration of 200 μg/ml. **B** Biofilm disruption assay using *A. vaginae* UG71161 biofilms. The different endolysin candidates, metronidazole and clindamycin were used in a final concentration of 200 μg/ml. OD_600_ readings are the average of biological triplicates plus/minus their standard deviation. The asterisks in figures indicate levels of significance. *p*-values < 0.05 are represented by *, *p-*values < 0.01 are represented by **, *p*-values < 0.001 are re*p*resented by *** and *p*-values < 0.0001 are represented by ****. No statistical differences are represented by ns. *p*-values for ANOVA and Tukey tests.

*G. vaginalis* is frequently found in multispecies biofilms along with *Atopobium vaginae*^20^, another pathobiont commonly linked to BV. It is thought that *G. vaginalis* initially forms a seeder biofilm layer, that can subsequently be synergistically colonized by *A. vaginae*^21^ and other BV associated pathobionts. We initially verified that as anticipated, the five selected endolysins had no activity when analysing against the *A. vaginae* UG71161 pathobiont (Table 4). However, given the evidence of polymicrobial biofilms in clinical cases of BV, we wanted to determine the impact of these endolysin candidates against polymicrobial biofilms. Biofilms cultivated using *G. vaginalis* ATCC14018 and *A. vaginae* UG71161 were subjected to biofilm disruption assays using concentrations of 200 μg/ml of the five endolysin candidates, metronidazole, and clindamycin (Fig. 7A). Additionally, disruption was assessed for *A. vaginae* monospecies biofilms (Fig. 7B) to determine the contribution of *A. vaginae*-specific activity of the candidates to overall polyspecies biofilm destruction. All five of the endolysin candidates could produce statistically significant biofilm disruption, whereas metronidazole and clindamycin did not exhibit significant biofilm elimination. Monospecies biofilms formed from *A. vaginae* UG71161 were not altered by endolysin or antibiotic treatment, reaffirming the specificity of the endolysin candidates and suggesting that anti-*Gardnerella* activity alone can disrupt polymicrobial biofilms.

## Discussion

Phage lytic proteins, such as endolysins, have excellent potential as novel treatments for bacterial infections, due to their specificity, their absence of reported bacterial resistance to date and their ability to disrupt bacterial biofilms^22,23^. Given the clear clinical need for novel BV therapeutics, and the association of growth with the dysbiosis, it was aimed to identify endolysins to target this problem pathobiont. After an extensive literature search, no publications describing the isolation of lytic phages infecting *G. vaginalis* species were found, however attempts to induce phage of lysogenic lifestyle have been recently attempted^24^. However, we hypothesized that prophages encoded in the genomes of different *G. vaginalis* strains represented an alternate source of phage lytic proteins targeting this pathobiont. Our study presents the global identification, characterization, and comparison of several distinct and diverse endolysin candidates derived from the prophage regions of selected datasets of diverse *Gardnerella* spp. genomes.

The use of phages and recombinant versions of their lytic proteins is a promising alternative to conventional antibiotic treatments in BV. Some phage endolysins are already progressing through clinical trials for other indications, and no adverse effects have been reported to date^25–27^. Prophages encoded in the genomes of different *G. vaginalis* strains are a potential good source to obtain phage lytic proteins targeting this pathogen^28^. This study presents for the first time the identification, characterization, and comparison of multiple different endolysin candidates that were encoded in prophages.

After an initial identification step, a panel of functionally diverse endolysin candidates were selected and their activity against *G. vaginalis* was assessed for clinical suitability. The potency and specificity of these candidates was demonstrated, and critically, their capacity to disrupt mono- and polyspecies *G. vaginalis* biofilms was observed. This biofilm disruption characteristic is especially encouraging: although several potential and combined causes of BV recurrence have been identified^29^ there is clear literature association between biofilm regrowth and recurrence of BV after antimicrobial and aseptic treatments^30–32^. Data derived from this study has further underlined the growing consensus that frontline BV therapies (such as metronidazole and clindamycin) are efficient in *G. vaginalis* biofilm prevention, but fail to eliminate pre-established biofilms^1,2,33,34^. These issues, and the wider antibiotic resistance crisis, are driving the need to search for alternative therapeutic options.

Other studies have searched for compounds (including peptides, antiseptics and surface-active agents) targeting *G. vaginalis* biofilms in vitro in an attempt to overcome the issue of symptom recurrence, which is so characteristic of BV associated-dysbiosis^20,33,34^. These compounds are not reported to share the host-specific bioactivities of our endolysin candidates as demonstrated by the selectivity studies outlined herein. A recent study has demonstrated bacteriolytic *G. vaginalis* biofilm disruption ex vivo by employing a single phage endolysin^16^ and some narrow structural homologues. We have expanded upon the results of this study by reporting a structurally diverse endolysin candidate library capable of potently disrupting *G. vaginalis* biofilms. We have further demonstrated how this activity was preserved when pathogens such as *A. vaginae* (currently classified as *Fannyhessa vaginae*) and *G. vaginalis* were co-cultured in a dual-species biofilm model.

It was additionally observed in this study that combinations of these endolysin candidates with metronidazole produced efficient biofilm disruption, even at very low concentrations. These results aligned with previous and contemporaneous reports showing the efficiency of endolysins in disrupting pre-established biofilms completely at low concentrations, comparable to the ones used in this study. Moreover, combining endolysins with traditionally used antibiotics and antimicrobial peptides^35–38^ results in better bactericidal activity. In this study it could be seen how some endolysins present a higher biofilm disruption efficiency when they are used in combination with metronidazole compared to when they are administered on their own. It is hypothesized that endolysins could, through their biofilm-disrupting capacity, liberate *G. vaginalis* cells from recalcitrant biofilms, rendering them more accessible to small molecule antimicrobials such as metronidazole in the vaginal environment. Metronidazole was selected for combined treatment with endolysins, and not clindamycin, as the results showed that other bacterial inhabitants in the vaginal environment were less susceptible to metronidazole than to clindamycin.

It has been previously reported that lactobacilli are not part of the polymicrobial biofilms associated with bacterial vaginosis, instead forming their own independent biofilms in other parts of the vaginal epithelium^12^. As a result, we contend that endolysin-mediated disruption of polymicrobial biofilms would not affect any commensal lactobacilli biofilm structures. This theory must be further evaluated by future in vivo studies.

The initial assessment of the potential of our candidates to disrupt *G. vaginalis* monospecies and *G. vaginalis/A. vaginae* biofilms was encouraging and demonstrated the potential for their development as clinical agents for the treatment of acute and recurrent bacterial vaginosis. The *G. vaginalis/A. vaginae* model was chosen as both species are highly associated with BV. However, further in vitro studies must be conducted against polyspecies biofilms which are more replicative of those found in BV in order to validate this hypothesis. These more complex biofilms must include the critical pathobiont *Prevotella bivia*, which has recently been hypothesized as a key component of BV biofilm development, growth and survival^39^ and as a potential effector (in concert with *G. vaginalis*) of BV-associated sequelae such as preterm birth through ascending uterine infection^40^. These more representative polyspecies biofilm studies are currently underway.

Further work also includes combining phage endolysins targeting *A. vaginae* (and other pathobionts associated with BV) with anti-*Gardnerella* endolysins in order to further enhance the disruption of BV-associated biofilms reported in this paper. We also intend to explore any potential adverse effects of these endolysins on epithelial cells or the eukaryotic hosts, although evidence from other endolysins that are currently under development indicates that endolysins are well tolerated by the host at therapeutic levels^41,42^.

The sequence and protein domain differences between our reported endolysins will also be further considered in the future design of mutant and/or chimeric derivatives and analogues of these candidates. It is well known that combining/altering catalytic or cell-binding domains of endolysins can result in candidates which are more potent, have different specificities and which are more drug-like/soluble. In the case of anti-*Gardnerella* endolysins, initial efforts at generating such derivatives through swapping of homologous domains have recently been shown^16^. Data obtained from this report will help guide us in further, more expansive candidate design. For example, in terms of biofilm disruption activity, the best results were obtained when employing CCB7.1 and CCB8.1. These two endolysins share the SH3b cell wall binding domain and also share LysA-like GH25 enzymatic active domains. This domain structure may be responsible for the particularly potent activity of these two candidates. We will continue to exploit the principles of endolysin engineering to refine this anti-*Gardnerella* activity. Creation of libraries of chimeric endolysins targeting BV-associated pathobionts is currently being explored.

**Fig. 8:**
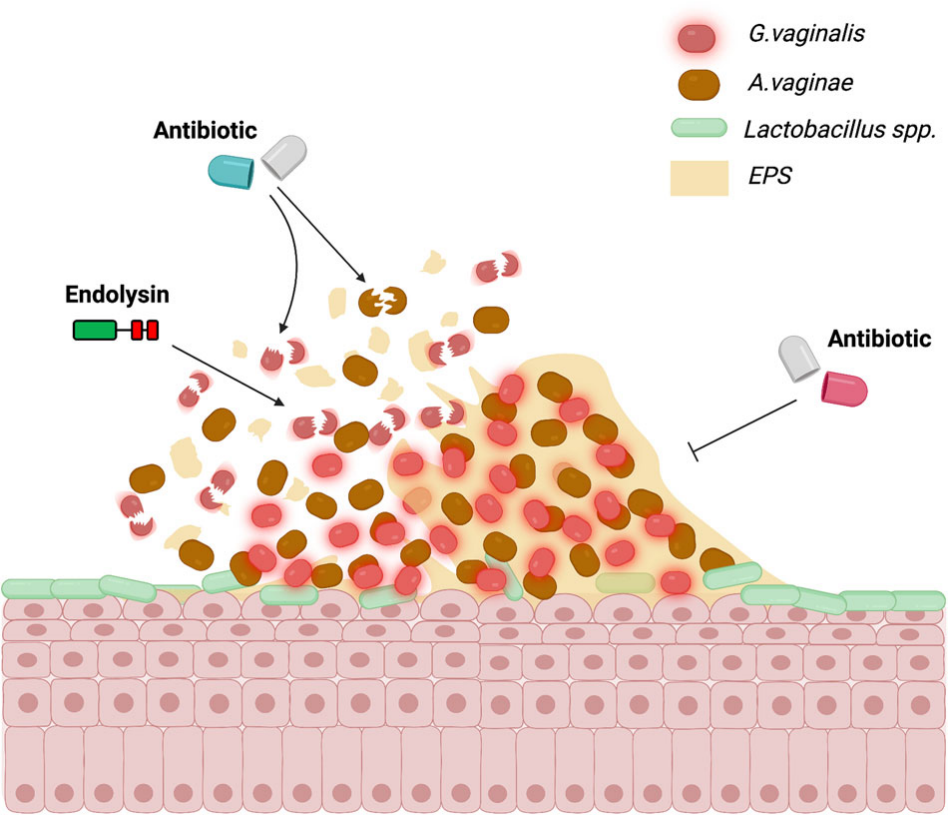
Proposed combined endolysin antibiotic therapeutic*:* The presence of recalcitrant biofilms typically mean that front line antibiotics are infective when treating *G. vaginalis* associated BV (right). Here we suggest that the lytic activity of phage endolysins leads to biofilm disruption and increased activity of front-line antibiotics (left) potentially leading to greater effective clearance and lower recurrence rates. Figure created using BioRender.

Finally, a key consideration in our future work will involve exploration of strategies for the effective delivery of these endolysins to the affected area, as oral therapy of a protein is unlikely to be an efficient route for a vaginal dysbiosis. In addition to formulation studies, efficacy and safety in vivo must be tested for these endolysin candidates, as well as exploring the safety implications of their possible use with other treatments such as metronidazole. However, based on the data outlined in this report, endolysins in combination with frontline therapy may be an effective strategy to tackle routine treatment failure and the high recurrence rate experienced by women suffering from BV (Fig. 8).

## Methods

### Bacterial strains and growth conditions

Clinically isolated strains of *Gardnerella vaginalis* UG860107 and *Atopobium vaginae* UG71161 were kindly provided by the Institute of Tropical Medicine, Antwerp in 2018 under an Material Transfer Agreement (MTA). *G. vaginalis* BUL001 is a morphologically distinct variant of *G. vaginalis* UG860107 isolated by the McCarthy lab. *G. vaginalis* UG860107 and *G. vaginalis* BUL001 were both typed as belonging to Genomospecies 1 based on *cpn60* sequence analysis according to Hill et al. (2019).^43^ (Supplementary Fig. 1). *G. vaginalis* ATCC 14018 was obtained from the Culture Collection, University of Gothenburg (CCUG). *G. vaginalis* ATCC14018, *G. vaginalis* UG860107, *G. vaginalis* strain and *A. vaginae* UG71161 were routinely grown on Columbia agar (Oxoid) supplemented with 5% horse blood (Thermo Scientific, UK). Single colonies from agar plates were inoculated into Brain Heart Infusion (BHI) broth (Sigma) supplemented with glucose (gBHI) or New York City III (NYC III) broth (1.5% (w/v) bactoproteose peptone no. 3, 0.5% (w/v) glucose, 0.24% (w/v) HEPES, 0.5% (w/v) NaCl, 0.38% (w/v) yeast extract, 10% (v/v) horse serum). *L. crispatus* CCUG42898, *L. gasseri* CCUG4409, *L. jensenii* CCUG35572 and *L. iners* CCUG38673 were cultivated on De Man, Rogosa and Shape (MRS) agar and broth (Sigma). All cultures were grown at 37 °C under anaerobic conditions using AnaeroGen Atmosphere Generation system (Thermo Scientific, UK) for 48 h.

*Escherichia coli* DH5α was used for routine cloning and plasmid maintenance. *E. coli* BL21(DE3) was used for expression of all recombinant endolysin proteins. For recombinant protein purification, *E. coli* BL21(DE3) harbouring an expression vector was cultured in Luria–Bertani (LB) broth containing 25 μg ml^−1^ kanamycin at 37 °C, at 200 rpm, until OD_600_ 0.5–0.6 was observed. Cultures were then induced with isopropyl β-d-1-thiogalactopyranoside (IPTG), before reducing incubation temperature to 16 °C, at 170 rpm, for 12 h.

### Endolysin discovery, phylogenetics and refactoring

A range of endolysin candidates were initially discovered from publicly available *Gardnerella* genome sequences. PHASTER^44^ (https://phaster.ca/) was used to interrogate these genome sequences to identify potential prophage regions. These regions were then analysed further using Blastp (https://blast.ncbi.nlm.nih.gov/Blast.cgi?PAGE=Proteins) to identify proteins within these prophage regions that contained putative endolysin domains. Multiple sequence alignments (MSA) of the endolysin candidate amino acid sequences were performed by Mega 11 (https://www.megasoftware.net/) using the MUSCLE algorithm with the following gap penalties: gap open: −2.9, gap extend: 0, hydrophobicity multiplier: 1.2. These parameters for opening and extending gap penalties are determinant on how frequent and short the gaps are in the sequence alignment. A maximum-likelihood phylogenetic tree was constructed from MSAs and bootstrapped with 500 iterations and was utilized to determine endolysin families CCB2-4 and CCB6-9. Endolysin homology models were constructed using SWISS MODEL (https://swissmodel.expasy.org/). A number of sequence diverse candidates lacking solvent exposed cysteine residues (as they can be modified if exposed to a solvent, altering the protein stability) from each family were selected for biochemical characterization at random^45^. Candidate endolysin gene sequences were refactored for expression in *Escherichia coli* using the GeneArt Designer software (Thermo Fisher Scientific) and synthesized and cloned into appropriate expression vectors by GenScript, USA. Each endolysin gene sequence comprised a removable N-terminal hexahistidine tag to aid solubility analysis and purification.

### Endolysin stability assays

Optimal buffer compositions for each recombinant endolysin were determined using the Durham Osmolyte Screen (MD1-120), pH screen (MD1-101) and salt screen (MD1102) as per manufacturer’s instructions (Molecular Dimensions, USA). Recombinant protein concentrations were normalized to 1 mg/ml and 10 μl aliquots were analysed. Quantitative increase in the intensity of sample fluorescence at 626 ± 14 nm was recorded, using the protein dye Spyro Orange (Thermo Fisher) and deconvoluted using QuantStudio6 flex (Thermo Fisher). Protein melt curves were visualized using matplotlib. pyplot and melt temperatures were calculated using NAMI^46^.

### Protein purification

*E. coli* BL21(DE3) cultures harbouring recombinant endolysins, grown as described in the section “Bacterial strains and growth condition”, were pelleted at 4000 × *g*, 4 °C, for 20 min and resuspended in chilled lysis buffer (PBS pH 7.4, 300 mM NaCl, DNase I (0.1 μg/mL) (New England Biolabs), before incubation on ice for 30 min. To obtain total cell lysate, cell suspensions were sonicated on ice as follows: amplitude 60%; 2 s on, 2 s off over 10 min for 2 cycles (Homogenizer UP100H, Hielscher). Following sonication, total cell lysates were centrifuged at 20,000 × *g*, 4 °C, for 40 min. Lysate supernatants were decanted and designated as soluble protein fractions. Pellets were washed and resuspended in lysis buffer and designated as insoluble protein fractions. Recombinant endolysin production and solubility were analysed by SDS–PAGE and Western Blot. SDS–PAGE analyses were performed according to the modification of Schagger and von Jagov method^47^ using the tris–tricine buffer system at 4% and 10% to stacking and separating gels, respectively. Gels were stained with Coomassie brilliant blue and visualized according to standard methods.

Following SDS–PAGE analysis, proteins were transferred to the PVDF membrane (0.45 μm; in Trans-Blot Turbo, Transfer Buffer, Bio-Rad). Membrane blocking was executed with TBS-T buffer supplemented with 5% non-fat milk. After washing in TBS-T, membranes were incubated overnight at 4 °C in TBS-T buffer containing H-3 IgG_1_ mouse monoclonal anti-His^6^ primary antibodies (SC-8036, Santa Cruz Biotechnology). Membranes were washed four times with TBS-T buffer before incubation with anti-mouse secondary antibodies (A9044, Sigma Aldrich) as above for 1 h at 20 °C. Following four washes with TBS-T, proteins were detected by colorimetric substrate (Pierce^TM^ 1—Step Ultra TMB Blotting Solution; Thermo Fisher Scientific). For recombinant endolysin stability assays, recombinant endolysins were purified by immobilized metal affinity chromatography (IMAC). In brief, soluble protein fractions were mixed with Ni-NTA resin (Qiagen) pre-equilibrated with lysis buffer supplemented with Complete™ Mini EDTA-free protease inhibitor cocktail (Roche, Basel, Switzerland). Resin:protein mixtures were incubated with gentle rotation (20 rpm) for 1.5 h at 4 °C before loading in appropriate gravity flow columns. Columns were subsequently washed with 10 column volumes (CV) of wash buffer (PBS pH 7.4, 300 mM NaCl, 20 mM Imidazole). Recombinant proteins were eluted in 1 CV (PBS pH 7.4, 300 mM NaCl, 20 mM Imidazole) and stored on ice.

Endolysins intended for bioactivity assays were further purified by size exclusion chromatography. IMAC fractions containing recombinant endolysins were pooled, dialyzed into Size-exclusion chromatography (SEC) buffer listed in Table 2 and concentrated using centrifugal filters. Concentrated endolysins were separated using a HiLoad 16/600 Superdex 75 pg or HiLoad 26/600 Superdex 75 pg column (GE Healthcare) pre-equilibrated with SEC buffer at flow rates of 1 and 2.6 mL/min, respectively. Protein elution was monitored at 280 nm and validated by SDS–PAGE (Supplementary Fig. 7). All blots were processed in parallel and derive from the same respective induction experiments.

Each recombinant endolysin was dialyzed into a bioactivity buffer system determined by endolysin protein stability assays, prior to bioactivity assays. Specifically, 200 mM MES (Sigma), 150 mM NaCl (Sigma), 4 mM dTT (Thermo Scientific, UK) and 10% glycerol (Fisher Scientific) pH 5.6 was utilized.

### MIC assays

Minimal inhibitory concentration (MIC) assays were performed according to the clinical laboratory standards institute CLSI^48^ guidelines, with minor modifications. In brief, serial 1:2 dilutions of each endolysin candidate, as well as metronidazole (Acros organic) and clindamycin (Acros organic) antibiotic controls were performed in the 96-well plates, using BHIg, MRS or NYCII broth, in final volumes of 100 μl. Turbid cultures of *G. vaginalis* ATCC14018, *L. crispatus* CCUG42898 *L. gasseri* CCUG4409, *L. jensenii* CCUG35572, *L. iners* CCUG38673 and *A. vaginae* UG71161 were diluted to OD600 0.1 in their growth media (BHIg, MRS or NYCIII), and 100 μl was aliquoted into the 96-well plates containing the different endolysin/antibiotic concentrations. Final concentrations ranged from 0.2 to 200 μg/ml. Plates were incubated anaerobically for 24 h at 37 °C using an AnaeroGen Atmosphere Generation system (Thermo Scientific, UK). After incubation, MICs were determined as the absence of visible growth. Each assay was performed in biological triplicate.

### Biofilm cultivation

Microtiter plate assays^49^ were used to analyse the biofilm formation of *G. vaginalis* ATCC14018, *G. vaginalis* UG860107, *G. vaginalis* BUL001 and *A. vaginae* UG71161. Monospecies biofilms were established using any of the three strains of *G. vaginalis* or *A. vaginae* UG71161, and dual-species biofilms were generated by co-culturing *G. vaginalis* ATCC14018 and *A. vaginae* UG71161. For monospecies *G. vaginalis* biofilms, cultures of *G. vaginalis* ATCC14018, *G. vaginalis* UG860107 and *G. vaginalis* BUL001 were prepared as described in the section “Bacterial strains and growth conditions” and diluted to an OD_600_ of 0.1 in BHIg. Prepared cultures were aliquoted in 200 µl volumes into wells of sterile bottomed flat 96-well microtiter plates (Sarstedt) and incubated for 48 h at 37 °C under anaerobic conditions. For *A. vaginae* UG71161 biofilms, the procedure was repeated using NYC III broth. For dual-species biofilms, *G. vaginalis* ATCC14018 and *A. vaginae* UG71161 were grown and diluted in NYC III broth to an OD_600_ of 0.1. 200 µl volumes of prepared *G. vaginalis* ATCC14018 bacterial solutions were dispensed into wells of sterile bottomed flat 96-well microtiter plates (Sarstedt) and then incubated for 24 h at 37 °C under anaerobic conditions. After 16 h, the contents of the wells were removed and 200 µl volumes of diluted *A. vaginae* UG71161 cultures were dispensed into the wells. The plates were incubated for an additional 24 h at 37 °C under anaerobic conditions. After this incubation period, plates were developed using either the Opentrons Robotic Liquid Handling platform or manually. In brief, the contents of the wells were removed, and the wells were washed three times with 200 μl of distilled water (dH_2_O). The biofilms were then stained with 200 μl of 1% crystal violet (Acros Organics) for 10 min at room temperature. The stain solution was removed, and the wells were gently washed as previously described. The plate was left to dry at room temperature, after which, 200 μl of 100% ethanol was added and the plate covered and left at room temperature overnight. The next day the OD_600_ of the 96-well plates was observed using a plate reader (SpectroStar, BMG LabTech).

### Resistance assays using serial passages

Endolysin candidates were assessed for their bacterial resistance potential through isolation of resistant colonies after passaging against sub-MIC endolysin concentrations, as previously described by Schuch et al.^50^ In brief, *G. vaginalis* ATCC14018 broth cultures were prepared as described in the section “Bacterial strains and growth conditions” and diluted to OD_600_ of 0.1 in BHIg containing 5 μg/ml final concentrations of individual endolysin candidates. Cultures were incubated for 24 h at 37 °C under anaerobic conditions. After this incubation period, cultures were centrifuged at 5000 × *g* for 5 min and pellets were resuspended in fresh BHIg containing individual endolysins at 10 μg/ml. This process was repeated for five passages with a doubling of endolysin concentration after each passage. 100 μl aliquots from the final passage were plated on Columbia blood agar plates with and without MIC concentration of each endolysin. Resistance frequencies were calculated as median number of mutants divided by the viable count^51^.

### Biofilm prevention assays

Cultures of *G. vaginalis* ATCC14018, *G. vaginalis* UG860107 and *G. vaginalis* BUL001 were diluted to an OD600 of 0.1. Serial 1:2 dilutions of the endolysin candidates, metronidazole, and clindamycin in BHIg were performed in sterile flat bottomed 96-well plates. Endolysins and antibiotic concentrations ranged from 0.2 to 200 μg/ml. 100 μl of prepared bacterial solutions were added to the different wells containing 100 μl of the different concentrations from a certain endolysin, antibiotic or 100 μl MES buffer as a control. Plates were incubated at 37 °C anaerobically for 48 h. After this incubation period, the plates were developed as described in the section “Monospecies and polyspecies biofilm disruption”.

### Biofilm disruption assays

Monospecies and polyspecies biofilms with *G. vaginalis* and *A. vaginae* were established as described in the section “Monospecies and polyspecies biofilm disruption”. Wells containing monospecies *G. vaginalis* biofilms were washed three times as described in the section “Monospecies and polyspecies biofilm disruption”, and then treated with 100 µl of each endolysin candidate, metronidazole, or clindamycin at final concentrations of 5, 50 and 200 µg/ml. Control wells were treated with endolysin buffer: 200 mM MES (Sigma) 150 mM NaCl (Sigma) 4 mM dTT (Thermo Scientific, UK) 10% Glycerol (Fisher Scientific) pH 5.6. These biofilms were also treated with combinations of endolysin candidates and metronidazole, leaving both compounds at final concentrations of 2.5 and 25 µg/ml. Plate wells containing *A. vaginae* UG71161 biofilms were also washed three times as described in the section “Monospecies and polyspecies biofilm disruption”, and then treated with 100 μl of endolysin candidates, metronidazole, and clindamycin at final concentrations of 200 µg/ml. Treated biofilm plates were anaerobically incubated at 37 °C for 48 h. After this incubation period, the contents of the wells were removed, and the plates were developed as described in the section “Monospecies and polyspecies biofilm disruption”.

### Reproducibility and statistical analysis

All set of the experiments were performed in triplicate using biological replicates. All comparisons between data were based on the mean of biological triplicates ± the standard deviation. ANOVA and subsequent Tukey tests were used to determine significance, with a *p*-value of <0.05 as the threshold to consider a result as statistically significant. Statistical analysis and graphs were generated using GraphPad Prism 9.1.0.

### Reporting summary

Further information on research design is available in the Nature Research Reporting Summary linked to this article.

## Supplementary information

Supplementary Material

Reporting Summary

## Data Availability

All relevant data are provided in the article or Supplementary information is available from the corresponding author upon reasonable request.
