# Supplementary material for: Identification and characterization of novel endolysins targeting Gardnerella vaginalis biofilms to treat bacterial vaginosis
Source: NPJ Biofilms Microbiomes. 2022 Apr 19;8:29. doi: 10.1038/s41522-022-00285-0 (PMC9018826; doi:10.1038/s41522-022-00285-0)
Supplement: Supplementary file 1 — Supplementary Material [file 41522_2022_285_MOESM1_ESM.pdf]

Supplementary Materials: Identification and characterization of novel endolysins targeting *Gardnerella vaginalis* biofilms to treat bacterial vaginosis.

Sara Arroyo-Moreno[1,2], Matthew Cummings[3], David B. Corcoran[3], Ronan R McCarthy[1*]

[1] Division of Biosciences, Department of Life Sciences, College of Health and Life Sciences, Brunel University London, Uxbridge, UB8 3PH, UK.
[2] Cork Institute of Technology, Cork, Cork, Ireland
[3] CC Biotech Ltd, London, United Kingdom

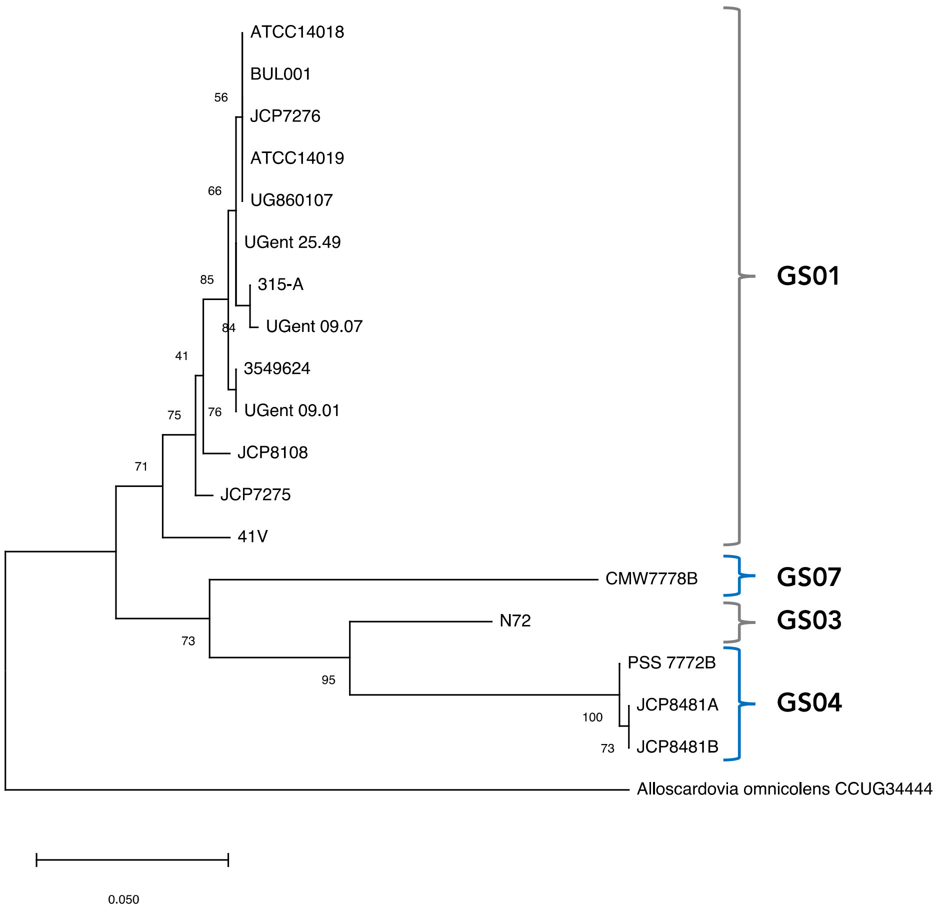

Supplementary Figure 1: Phylogenetic analysis of *Gardnerella vaginalis* Cpn60 chaperone DNA sequence. *G. vaginalis* UG860107 and BUL001 clade with clade C Gardnerella spp., or GS01 Gardnerella genomospecies as defined by Jayaprakash, Schellenberg and Hill (2012) and Potter *et. al.* (2019), respectively. Cpn60 sequences were retrieved from CpnDB (https://www.cpndb.ca/) on November 2021, or sequenced directly as previously described (3). Multiple sequence alignments and phylogenetic trees were constructed in Mega as described in Materials and Methods. Cpn60 gene sequences from *Alloscardova omnicolens* CCUG34444 was used as an outgroup. GS: genomospecies.

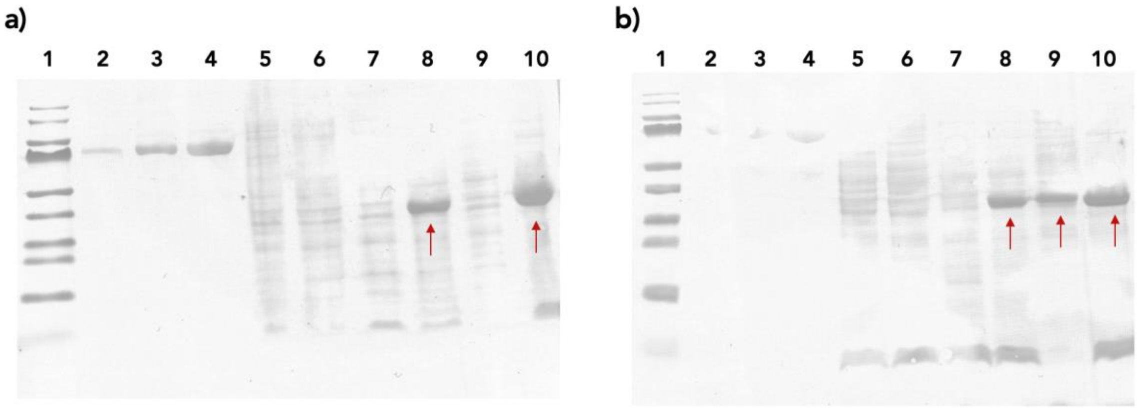

Supplementary Figure 2: A) Solubility of recombinant His^{6}-CCB2M84_97 in PBS pH 7.4 determined by Western Blot. Lane 1: molecular weight ladder. Protein standard sizes are shown in kDa. Lane 2: conalbumin (2 µg), Lane 3: conalbumin (5 µg), Lane 4: conalbumin (10 µg), Lane 5: host control - *E. coli* BL21(DE3) cell lysate, Lane 6: cell lysate from *E. coli* BL21(DE3) induced with IPTG, Lane 7: uninduced *E. coli* BL21(DE3) pCCB2M84_97 cell lysate, Lane 8: *E. coli* BL21(DE3) pCCB2M84_97 total cell lysate. Lane 9: *E. coli* BL21(DE3) pCCB2M84_97 soluble protein fraction. Lane 10: *E. coli* BL21(DE3) pCCB2M84_97 insoluble protein fraction. **B)** Solubility of recombinant His^{6}-CCB2M87_2 in PBS pH 7.4 determined by Western Blot. Lanes 1 through 6 are as described in A. Lane 7: uninduced *E. coli* BL21(DE3) pCCB2M87_2 cell lysate, Lane 8: *E. coli* BL21(DE3) pCCB2M87_2 total cell lysate. Lane 9: Soluble protein fraction. Lane 10: Insoluble protein fraction. The molecular weights of His^{6}-CCB2M84_97 and His^{6}-CCB2M87_2 are 35.94 and 35.95 kDa, respectively.

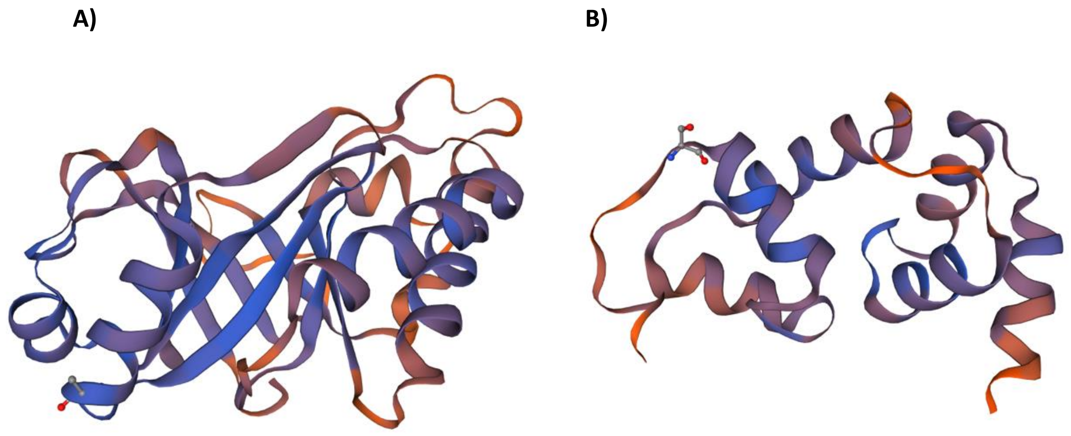

Supplementary Figure 3: **A)** CCB2M87_2 enzymatic active domain homology model constructed using an endolysin from the *Clostridium perfringens* phage phiSM101 (PDB: 4krt.2) as a template. Ala53 is shown as grey sticks and balls. **B)** CCB2M87_2 cell wall binding domain homology model constructed using PDB: 5i8l.1 as a template. Asparagine 233 is shown as grey sticks and balls.

A)

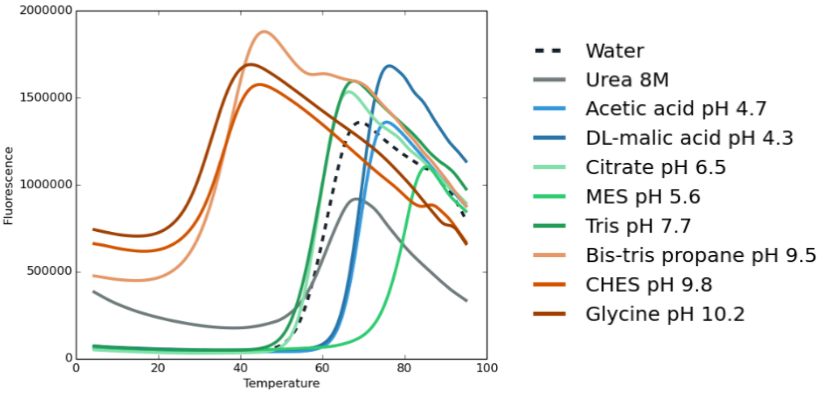
2000000
1500000
1000000
500000
0
Fluorescence
0
20
40
60
80
100
Temperature
Water
Urea 8M
Acetic acid pH 4.7
DL-malic acid pH 4.3
Citrate pH 6.5
MES pH 5.6
Tris pH 7.7
Bis-tris propane pH 9.5
CHES pH 9.8
Glycine pH 10.2

B)

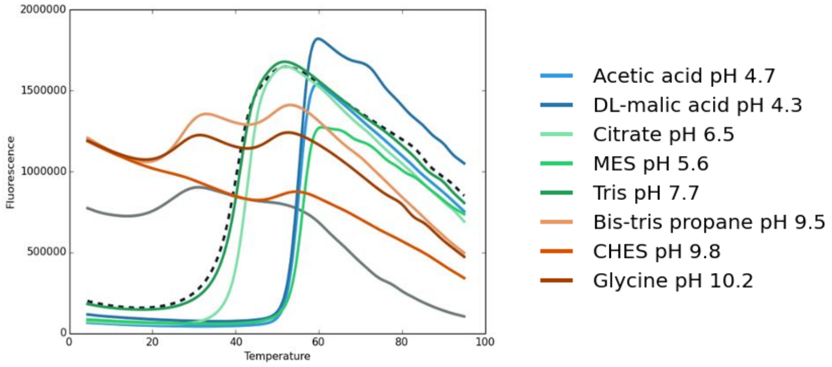
2000000
1500000
1000000
500000
0
Fluorescence
0
20
40
60
80
100
Temperature
Acetic acid pH 4.7
DL-malic acid pH 4.3
Citrate pH 6.5
MES pH 5.6
Tris pH 7.7
Bis-tris propane pH 9.5
CHES pH 9.8
Glycine pH 10.2

C)

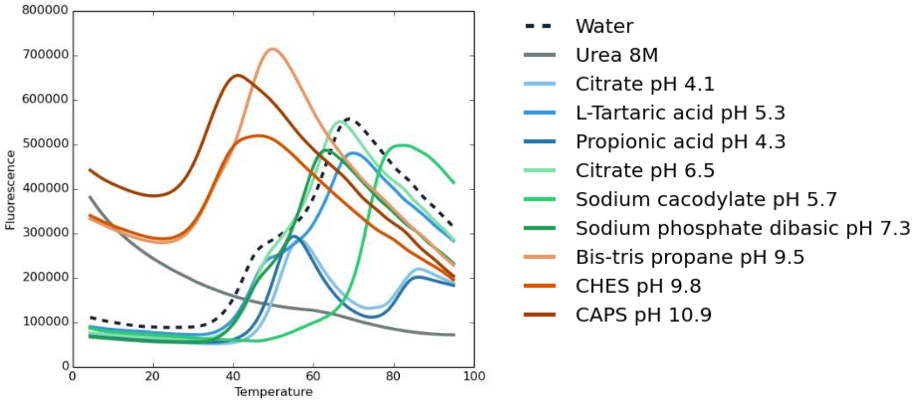
800000
700000
600000
500000
400000
300000
200000
100000
0
Fluorescence
0
20
40
60
80
100
Temperature
Water
Urea 8M
Citrate pH 4.1
L-Tartaric acid pH 5.3
Propionic acid pH 4.3
Citrate pH 6.5
Sodium cacodylate pH 5.7
Sodium phosphate dibasic pH 7.3
Bis-tris propane pH 9.5
CHES pH 9.8
CAPS pH 10.9

D)

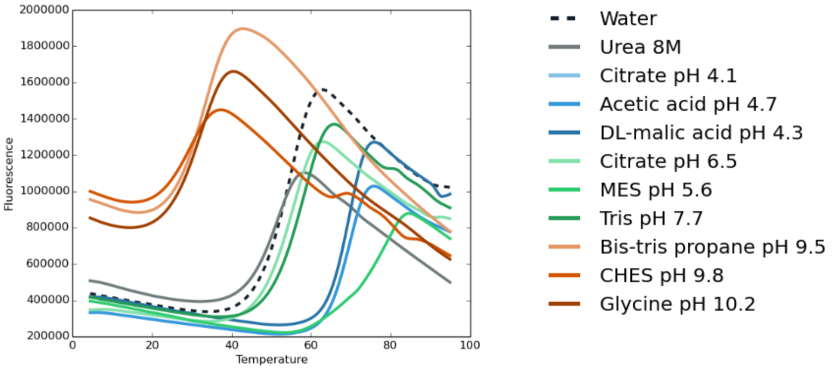
Fluorescence
Temperature
Water
Urea 8M
Citrate pH 4.1
Acetic acid pH 4.7
DL-malic acid pH 4.3
Citrate pH 6.5
MES pH 5.6
Tris pH 7.7
Bis-tris propane pH 9.5
CHES pH 9.8
Glycine pH 10.2

E)

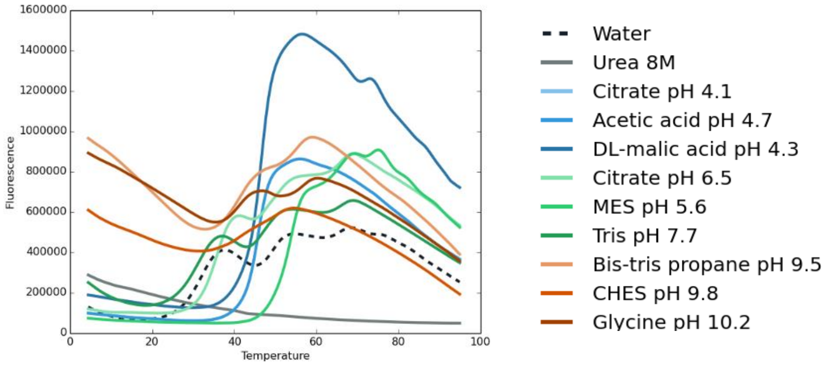
Fluorescence
Temperature
Water
Urea 8M
Citrate pH 4.1
Acetic acid pH 4.7
DL-malic acid pH 4.3
Citrate pH 6.5
MES pH 5.6
Tris pH 7.7
Bis-tris propane pH 9.5
CHES pH 9.8
Glycine pH 10.2

F)

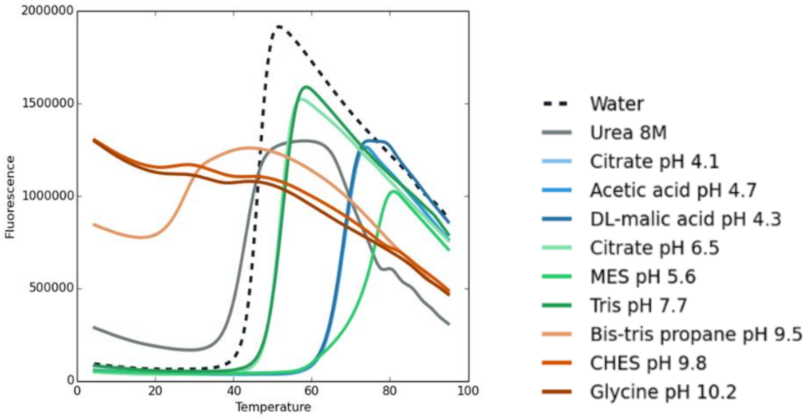
Fluorescence
Temperature
Water
Urea 8M
Citrate pH 4.1
Acetic acid pH 4.7
DL-malic acid pH 4.3
Citrate pH 6.5
MES pH 5.6
Tris pH 7.7
Bis-tris propane pH 9.5
CHES pH 9.8
Glycine pH 10.2

G)

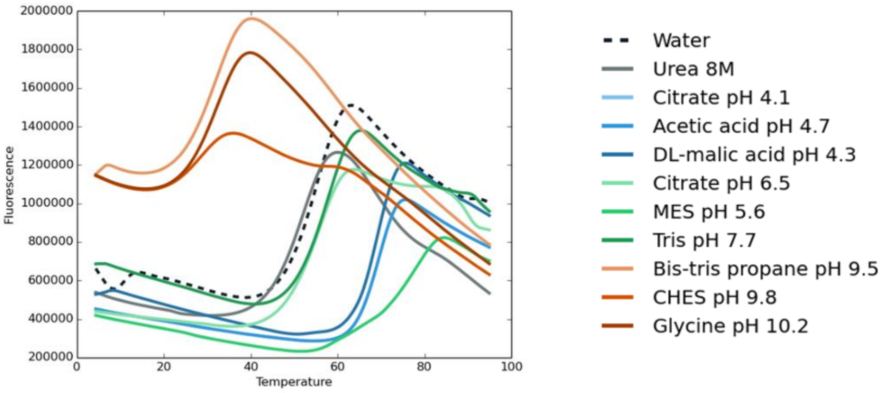
Fluorescence
Temperature
2000000
1800000
1600000
1400000
1200000
1000000
800000
600000
400000
200000
0
20
40
60
80
100
Water
Urea 8M
Citrate pH 4.1
Acetic acid pH 4.7
DL-malic acid pH 4.3
Citrate pH 6.5
MES pH 5.6
Tris pH 7.7
Bis-tris propane pH 9.5
CHES pH 9.8
Glycine pH 10.2

H)

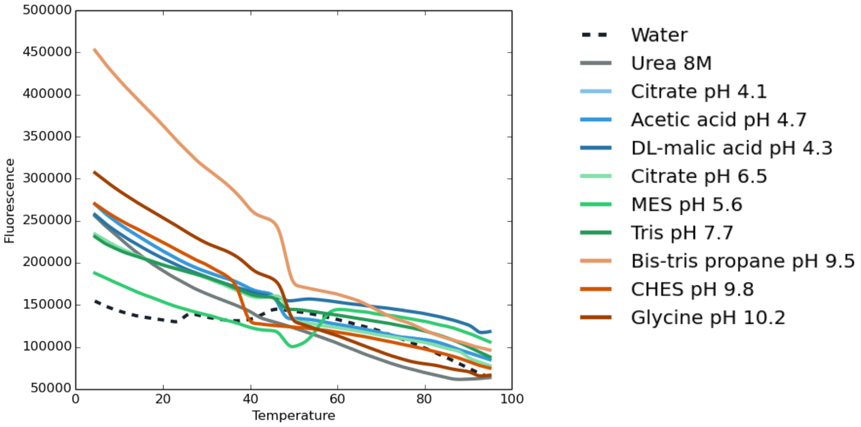
Fluorescence
Temperature
500000
450000
400000
350000
300000
250000
200000
150000
100000
50000
0
20
40
60
80
100
Water
Urea 8M
Citrate pH 4.1
Acetic acid pH 4.7
DL-malic acid pH 4.3
Citrate pH 6.5
MES pH 5.6
Tris pH 7.7
Bis-tris propane pH 9.5
CHES pH 9.8
Glycine pH 10.2

I)

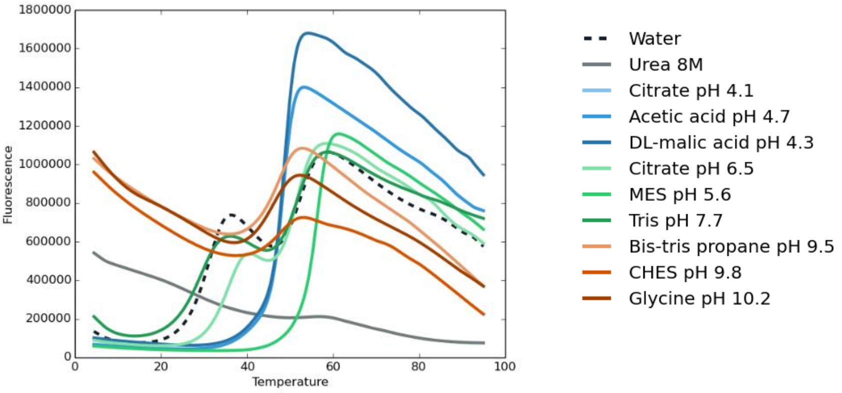
Fluorescence
Temperature
1800000
1600000
1400000
1200000
1000000
800000
600000
400000
200000
0
20
40
60
80
100
Water
Urea 8M
Citrate pH 4.1
Acetic acid pH 4.7
DL-malic acid pH 4.3
Citrate pH 6.5
MES pH 5.6
Tris pH 7.7
Bis-tris propane pH 9.5
CHES pH 9.8
Glycine pH 10.2

J)

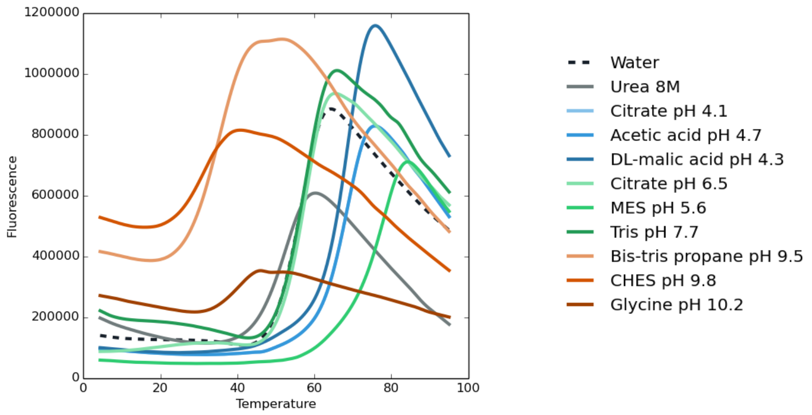

Supplementary Figure 4 A) Thermal shift analysis of CCB2M94_8 in varying buffer systems. Melt temperatures are as follows: Water (Tm 59.88ºC), Urea 8 m (Tm 59.21ºC), Citrate pH 4.1 (Tm 68.33ºC), Acetic acid pH 4.7 (Tm 68.42ºC), DL-Malic acid pH 4.3 (Tm 68.66), Citrate pH 6.5 (Tm 58.33ºC), MES pH 5.6 (Tm 77.76 ºC), Tris pH 7.7 (Tm 58.26), Bis-tris propane pH 9.5 (Tm 35.38ºC), CHES pH 9.8 (Tm 34.29), Glycine pH 10.2 (Tm 31.81). **B)** Thermal shift analysis of CCB7.1 in varying buffer systems. Water (Tm 39.42ºC), Urea 8 M (23.61ºC), Citrate pH 4.1 (Tm 54.72ºC), Acetic acid pH 4.7 (Tm 54.59ºC), DL-Malic acid pH 4.3 (Tm 54.83ºC), Citrate pH 6.5 (Tm 42.45ºC), MES pH 5.6 (Tm 55.51ºC), Tris pH 7.7 (Tm 40.27ºC), Bis-tris propane pH 9.5 (Tm 26.34ºC), CHES pH 9.8 (Tm 50.16ºC), Glycine pH 10.2 (Tm 25.53ºC) **C)** Thermal shift analysis of CCB8.1 in varying buffer systems. Melt temperatures are as follows: Water (Tm 53.23ºC), Urea 8 M (Not detected), Citrate pH 4.1 (Tm 50.23ºC), L-Tartaric acid pH 5.3 (Tm 53.95ºC), Propionic acid pH 4.3 (Tm 48.9ºC), Citrate pH 6.5 (Tm 71.7ºC), Sodium cacodylate pH 5.7 (Tm 71.7ºC), Sodium phosphate dibasic pH 7.3 (Tm 51.13ºC), Bis-tris propane pH 9.5 (Tm 39.39ºC), CHES pH 9.8 (Tm 34.76ºC), CAPS pH 10.9 (Tm 32.75ºC). Fluorescence is measure in arbitrary units (au). **D)** Thermal shift analysis of CCB2.2 in varying buffer systems. Water (Tm 52.9ºC), A4: Urea 8 m (Tm 49.5ºC), A4: Citrate pH 4.1 (Tm 68.2ºC), A8: Acetic acid pH 4.7 (Tm 67.79ºC), DL-Malic acid pH 4.3 (Tm 68.36), C3: Citrate pH 6.5 (Tm 53.3ºC), MES pH 5.6 (Tm 73.4 ºC), G1: Tris pH 7.7 (Tm 56.06), G12: Bis-tris propane pH 9.5 (Tm 31.94ºC), H6: CHES pH 9.8 (Tm 28.22), H9 Glycine pH 10.2 (Tm 30.56) **E)** Thermal shift analysis of CCB4.1 in varying buffer systems. Water (Tm 30.53ºC), Urea 8 M (not detected), Citrate pH 4.1 (Tm 45.51ºC), Acetic acid pH 4.7 (Tm 46.52ºC), DL-Malic acid pH 4.3 (Tm 46.14ºC), Citrate pH 6.5 (Tm 41.48ºC), MES pH 5.6 (Tm 54.07ºC), Tris pH 7.7 (Tm 36.93ºC), Bis-tris propane pH 9.5 (Tm 44.97ºC), CHES pH 9.8 (Tm 44.12), Glycine pH 10.2 (Tm 45.15). **F)** Thermal shift analysis of CCB2M90_2 in varying buffer systems. Water (Tm 45.8ºC), Urea 8 m (Tm 42.64ºC), Citrate pH 4.1 (Tm 67.21ºC), Acetic acid pH 4.7 (Tm 67.16ºC), DL-Malic acid pH 4.3 (Tm 67.29ºC), C3: Citrate pH 6.5 (Tm 51.27ºC), MES pH 5.6 (Tm 72.42 ºC), Tris pH 7.7 (Tm 51.8ºC), Bis-tris propane pH 9.5 (Tm 28.02ºC), CHES pH 9.8 (Tm 24.63ºC), Glycine pH 10.2 (Tm 41.17ºC) **G)** Thermal shift analysis of CCB2.4 in varying buffer systems. Water (Tm 53.78ºC), Urea 8 M (Tm 50.13ºC), Citrate pH 4.1 (Tm 68.37ºC), Acetic acid pH 4.7 (Tm 67.58ºC), DL-Malic acid pH 4.3 (Tm 68.1ºC), Citrate pH 6.5 (Tm 53.74ºC), MES pH 5.6 (Tm 72.7ºC), Tris pH 7.7 (Tm 56.06ºC), Bis-tris propane pH 9.5 (Tm 30.34ºC), CHES pH 9.8 (Tm 27.36), Glycine pH 10.2 (Tm 30.64) **H)** Thermal shift analysis of CCB3.2 in varying buffer systems, analysis of starting material shows CCB3.2 to be denatured. **I)** Thermal shift analysis of CCB4.2 in varying buffer systems. Water (Tm 52.29ºC, second peak), Urea 8 M (52.69ºC), Citrate pH 4.1 (Tm 47.36ºC), Acetic acid pH 4.7 (Tm 48.82ºC), DL-Malic acid pH 4.3 (Tm 47.84ºC), Citrate pH 6.5 (Tm 44.24ºC), MES pH 5.6 (Tm 55.12ºC), Tris pH 7.7 (Tm 51.61ºC), Bis-tris propane pH 9.5 (Tm 46.04ºC), CHES pH 9.8 (Tm 46.58ºC), Glycine pH 10.2 (Tm 45.37ºC). **J)** Thermal shift analysis of CCB230b in varying buffer systems. Water (Tm 55.33ºC), Urea 8 M (50.62ºC), Citrate pH 4.1 (Tm 66.2ºC), Acetic acid pH 4.7 (Tm 66.44ºC), DL-Malic acid pH 4.3 (Tm 66.28ºC), Citrate pH 6.5 (Tm 56.45ºC), MES pH 5.6 (Tm 73.54ºC), Tris pH 7.7 (Tm 56.42ºC), Bis-tris propane pH 9.5 (Tm 34.3ºC), CHES pH 9.8 (Tm 30.2ºC), Glycine pH 10.2 (Tm 38.83ºC).

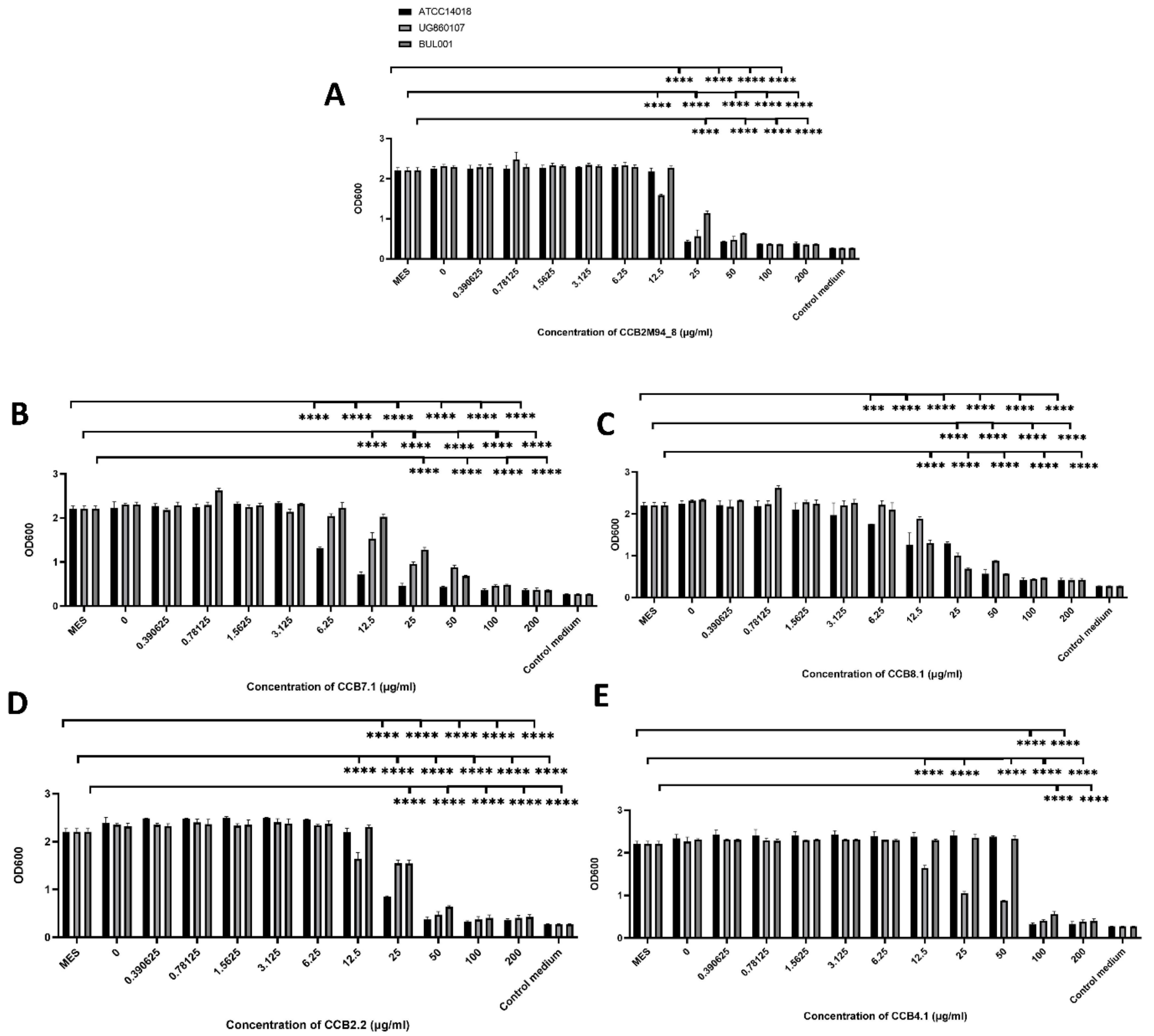

Supplementary Figure 5. ***Endolysin Biofilm Prevention Activity:*** Biofilm prevention assay using *G. vaginalis* ATCC14018, *G. vaginalis* UG860107 and *G. vaginalis* BUL001 cultures. Final concentrations of (A) CCB2M94_8, (B) CCB7.1, (C) CCB8.1, (D) CCB2.2 or (E) CCB4.1, from 0.39 to 200 µg/µl were used to prevent biofilms. OD_{600} readings are the average of biological triplicates. The asterisks in figures indicate levels of significance. p-values < 0.05 are represented by *, *p*-values < 0.01 are represented by **, *p*-values < 0.001 are represented by *** and *p*-values < 0.0001 are represented by ****. No statistical differences are represented by ns.

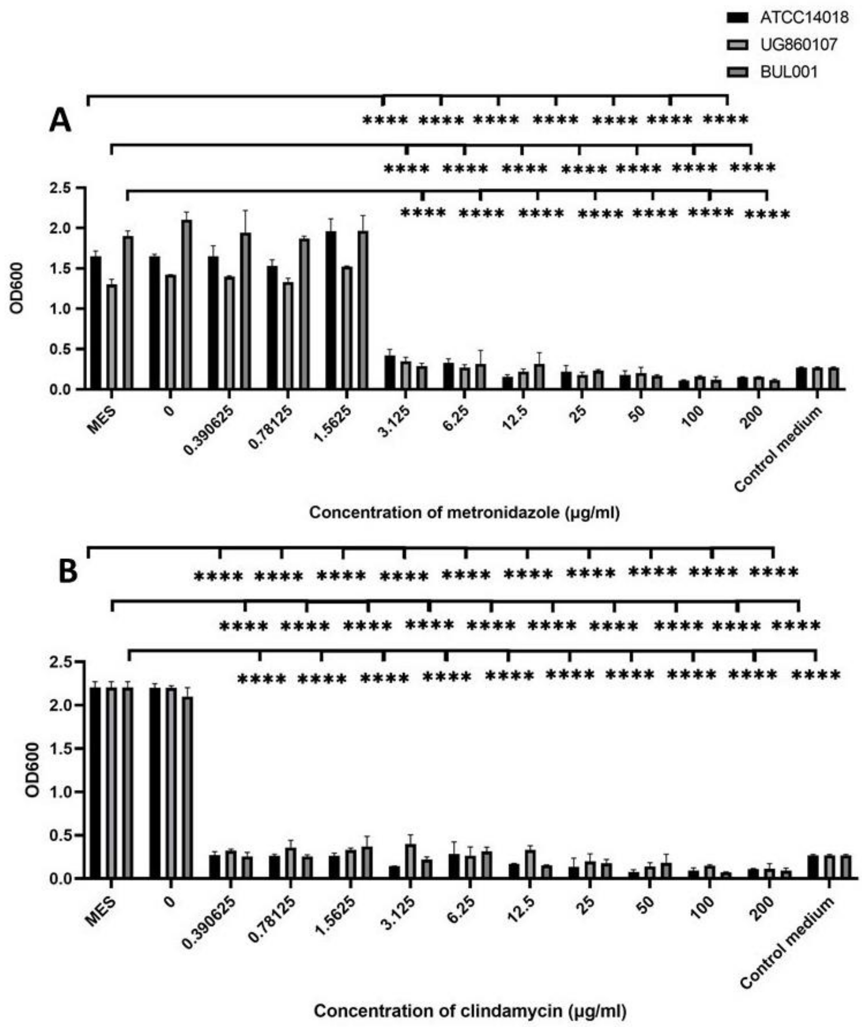

Supplementary Figure 6. Antibiotic Biofilm Prevention Activity: Biofilm prevention assays using G. vaginalis ATCC14018, G. vaginalis UG860107 and G. vaginalis BUL001 cultures. Final concentrations of (A) metronidazole or (B) clindamycin from 0.39 to 200 µg/µl, were used to disrupt biofilms. OD600 readings are the average of biological triplicates. The asterisks in figures indicate levels of significance. p-values < 0.05 are represented by *, p-values < 0.01 are represented by **, p-values < 0.001 are represented by *** and p-values < 0.0001 are represented by ****. No statistical differences are represented by ns.

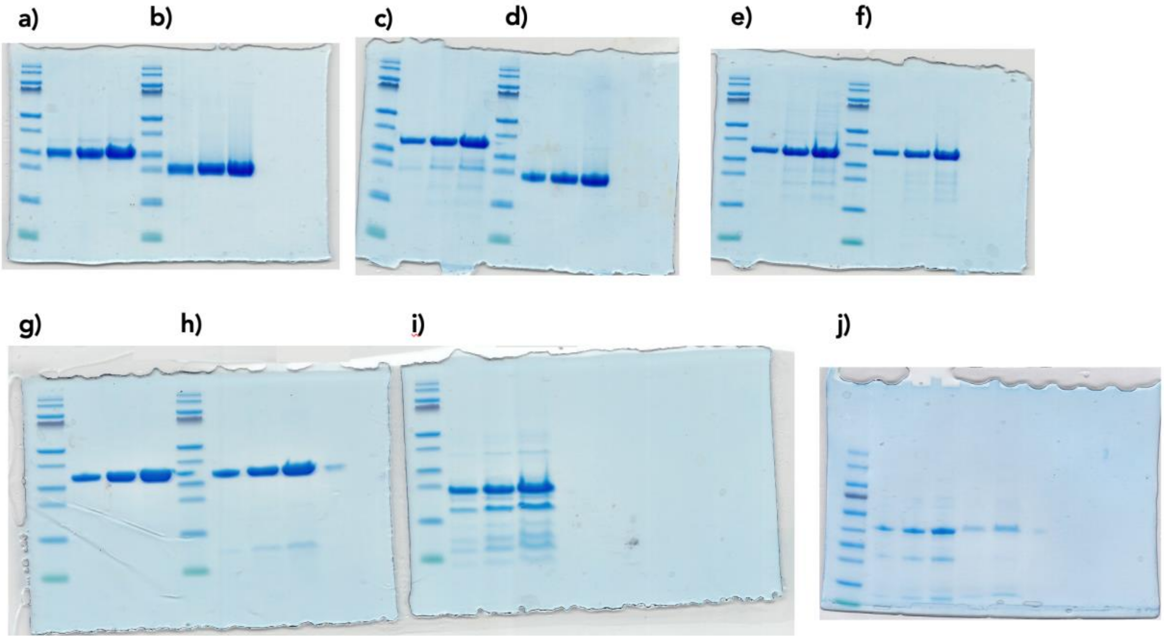

Supplementary Figure 7: Purified recombinant endolysin proteins: **A)** CCB2.2 (30 kDa) **B)** CCB2.4 (25 kDa) **C)** CCB8.1 (36 kDa) **D)** CCB4.2 (25 kDa) **E)** CCB7.1 (36 kDa) **F)** CCB4.1 (38 kDa) **G)** CCB2M90_2 (36 kDa) **H)** CCB2M94_8 (36 kDa) **I)** CCB3.2 **J)** CCB230b (40 kDa) Molecular weight ladder values from top to bottom are as follows: 170, 130, 95, 72, 55, 43, 34, 26, 17, 10 kDa. Each purified endolysin candidates was visualised at increasing concentrations (2, 5 and 10 µg).

Supplementary Table 1: Distribution of CCB family endolysin gene sequences across Gardnerella genomospecies. Genomospecies are as outlined by Potter, Burnham and Dantas (2019).

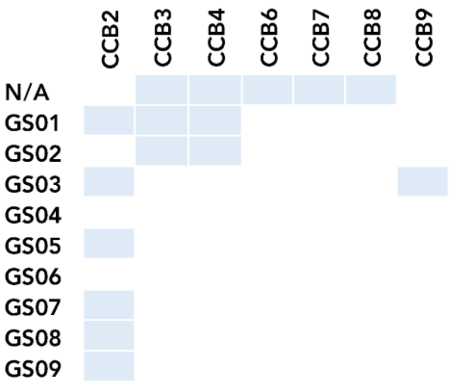

Supplementary Table 2. Protein accession numbers for parent sequence of each fully characterised endolysin candidate. Accession numbers/source material for homologues are not detailed.

Candidate Name	Accession	Source Material	Genomospecies*
CCB2M94_8	Derived from WP_116712567.1	*G. vaginalis* NR010	GS09
CCB2.2	Derived from WP_116691955.1	*G. vaginalis* N165	GS01
CCB4.1	Derived from WP_075038579.1	*G. vaginalis* ATCC 49145	GS01
CCB7.1	Derived from WP_102168908.1	*G. vaginalis* UMB0388	N/A
CCB8.1	Derived from PMC27097.1	*G. vaginalis* UMB0388	N/A

*Genomospecies are as outlined by Potter, Burnham and Dantas (2019). N/A is stated where organisms fall outside of this taxonomic classification. Notable examples include a CCB4 family endolysin identified in *Gardnerella vaginalis* FDAARGOS_568, and CCB8 and CCB7 candidates which were isolated from *Gardnerella vaginalis* UMB0388. GenBank nucleotide entries for the latter strain indicate this organism to be a *Gardnerella vaginalis* isolate (accession: PNGN00000000.1), however the exact provenance of this DNA has recently been questioned (4, 5).

References:

1. Jayaprakash, T. P., Schellenberg, J. J. and Hill, J. E., Resolution and Characterization of Distinct cpn60-Based Subgroups of Gardnerella vaginalis in the Vaginal Microbiota *PLoS one* **7** e43009 (2012) DOI: 10.1371/journal.pone.0043009.

2. Potter, R.F., Burnham, C., Dantas, G., In Silico Analysis of Gardnerella Genomospecies Detected in the Setting of Bacterial Vaginosis. *Clin. Chem.* **65** 1375-1387 (2019) DOI: 10.1373/clinchem.2019.305474

3. Hill, J. E., Town, J. R., Hemmingsen, S. M., Improved template representation in cpn60 polymerase chain reaction (PCR) product libraries generated from complex templates by application of a specific mixture of PCR primers. *Environ. Microbiol.* **8** 741-746 (2006) DOI: 10.1111/j.1462-2920.2005.00944.x

4. Tortelli, B. A., Lewis, A. L., Fay, J. C. The structure and diversity of strain level variation in vaginal bacteria. *Microb. Genom.* **7** mgen000543 (2021) DOI: 10.1099/mgen.0.000543

5. Tarracchini, C., Lugli, G. A., Mancabelli, L., Milani, C., Turroni, F., Ventura., M. Assessing the Genomic Variability of Gardnerella vaginalis through Comparative Genomic Analyses: Evolutionary and Ecological Implications. *Appl. Environ. Microbiol.* **87** e02188-20 (2020) DOI: 10.1128/AEM.02188-20
